# Demography and environment modulate the effects of genetic diversity on extinction risk in a butterfly metapopulation

**DOI:** 10.1073/pnas.2309455121

**Published:** 2024-08-08

**Authors:** Michelle F. DiLeo, Abhilash Nair, Marty Kardos, Arild Husby, Marjo Saastamoinen

**Affiliations:** ^a^Research Centre for Ecological Change, Organismal and Evolutionary Biology Research Programme, Faculty of Biological and Environmental Sciences, University of Helsinki, Helsinki 00014, Finland; ^b^Wildlife Research and Monitoring Section, Ontario Ministry of Natural Resources, Peterborough, ON K9L 1Z8, Canada; ^c^Conservation Biology Division, Northwest Fisheries Science Center, National Marine Fisheries Service, National Oceanic and Atmospheric Administration, Seattle, WA 98112; ^d^Evolutionary Biology, Department of Ecology and Genetics, Uppsala University, Uppsala 75236, Sweden

**Keywords:** heterozygosity, fitness, extirpation, conservation genetics, inbreeding

## Abstract

Linking genetic diversity to extinction is a common goal in genomic studies. Recently, a debate has arisen regarding the importance of genetic variation in conservation as some studies have failed to find associations between genome-wide genetic diversity and extinction risk. However, only rarely are genetic diversity and fitness measured together in the wild, and typically demographic history and environment are ignored. It is therefore difficult to infer whether a lack of an association is real or obscured by confounding factors. To address these shortcomings, we analyzed genetic data from 7,501 individuals with extinction data from 279 meadows and mortality of 1,742 larval nests in a butterfly metapopulation. We found a strong negative association between genetic diversity and extinction when considering only heterozygosity in models. However, this association disappeared when accounting for ecological covariates, suggesting a confounding between demography and genetics and a more complex role for heterozygosity in extinction risk. Modeling interactions between heterozygosity and demographic variables revealed that associations between extinction and heterozygosity were context-dependent. For example, extinction declined with increasing heterozygosity in large, but not currently small populations, although negative associations between heterozygosity, extinction, and mortality were detected in small populations with a recent history of decline. We conclude that low genetic diversity is an important predictor of extinction, predicting >25% increase in extinction beyond ecological factors in certain contexts. These results highlight that inferences about the importance of genetic diversity for population viability should not rely on genomic data alone but require investments in obtaining demographic and environmental data from natural populations.

## Significance

Genome-wide genetic diversity is often used to help assess extinction risk. However, some studies have found that genetic diversity does not always predict population viability. This raises the question: under what conditions do we expect to find associations between genetic diversity and extinction in nature? By linking genetic data from thousands of butterflies with ecological data from hundreds of local populations, we find that associations between genetic diversity and extinction are strong but context dependent. Importantly, drawing accurate conclusions required proper control of the confounding influences of demography and dispersal. This highlights that inferences about the importance of genetic diversity should not rely on genomic data alone but require strong investments in obtaining demographic and environmental data from natural populations.

The role of genetic variation in population persistence has gained renewed interest over the last few years, as more genomes from critically endangered or even extinct species are sequenced (1–6). While theory predicts that extinction risk should be highest for small populations that have low genetic diversity (7, 8), some recent empirical studies have failed to find the expected relationship between genetic diversity and extinction risk. Instead, these studies (although few) show that populations have persisted despite small sizes and/or high levels of inbreeding (4) or alternatively have gone extinct or declined with no indication of genomic erosion (1). This has led some to question the value of neutral or genome-wide genetic diversity in conservation, instead emphasizing that reintroductions and rescue should focus on managing populations for adaptive or deleterious variation (9, 10). Others have argued that we can understand these conflicting results by recognizing that wild populations may not be in mutation-drift-selection equilibrium due to environmental and/or demographic stochasticity (11) and that exceptions to the rule may reflect survivorship bias (12, 13). Thus, the question remains, under what natural conditions do we expect to find a negative association between genetic diversity and extinction risk? Answering this question remains extremely challenging, as it requires linking genetic variation, fitness, and extinction risk across many populations in their natural environments—data that are very hard to obtain.

Nonetheless, empirical studies have detected the expected negative relationship between fitness and genome-wide genetic diversity (14–17), and some studies even between genetic diversity and population dynamics (2, 18). There are however several reasons why we may not observe the expected relationship between genetic diversity and extinction risk in natural populations. For example, a lag is expected between a population decline and subsequent loss of genetic diversity. The time required for genetic diversity to erode to the extent it can negatively impact fitness and thus increase extinction risk will vary depending on factors such as the effective population size, generation time, mutation rate, selection pressure, distribution of fitness effects, and dispersal (11, 19–22). Sustained population declines are expected to lead to extinction in small populations eventually, whereas short-duration bottlenecks might not lead to severely reduced fitness, and negative fitness effects can even be reversed if there is rescue through immigration or if environmental conditions turn favorable (23). Bottlenecks can also happen in the presence of inbreeding depression-by-environment interactions leading to reduced genetic diversity and increased inbreeding, but with no apparent effect on fitness (24). For example, there is good evidence that inbreeding depression is more severe in stressful rather than benign environments (25–27) although the statistical power to detect this can differ between field and lab studies (28). Even under strong inbreeding depression, populations can persist despite mortality of inbred individuals if inbred individuals are replaced with individuals of higher fitness by soft selection reducing the frequency of deleterious alleles within demes rather than across demes (29, 30). Indeed, soft selection might be particularly dominant in spatially structured populations, where spatial heterogeneity in the environment and in selection pressures can maintain diversity and facilitate rescue across populations connected by dispersal (31, 32). Therefore, testing hypotheses about the importance of genetic diversity for persistence must carefully account for confounding impacts of demography and environment—something that most studies do not consider (1, 3–5).

**Fig. 1.**
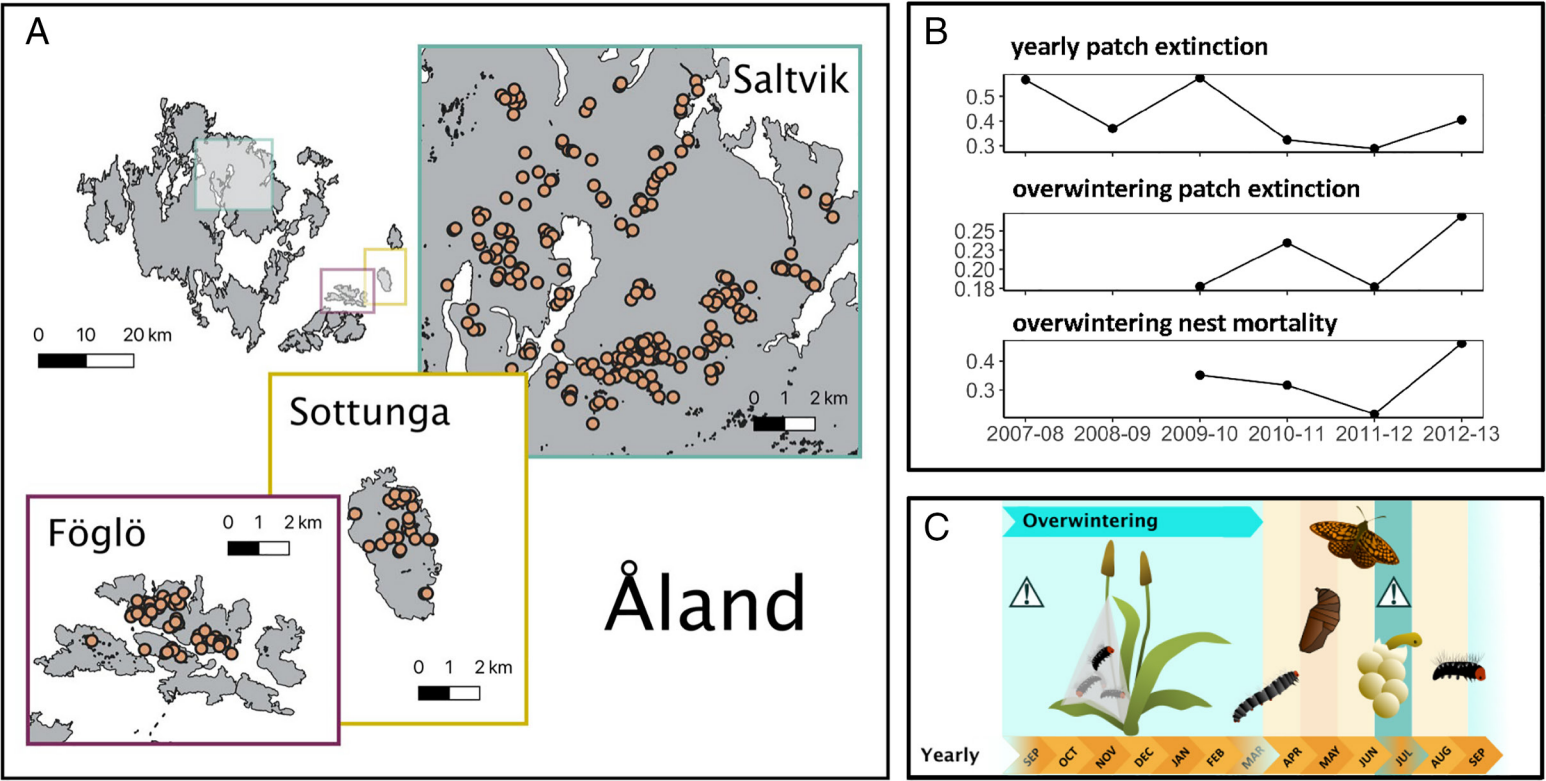
Map of three study areas in Åland, Finland (*A*), trends in extinction and mortality (*B*), and lifecycle of *M. cinxia* (*C*). Larvae were sampled from three regions: Saltvik, Föglö, and Sottunga. Habitat patches in (*A*) are shown in black and sampled populations are indicated by circles. Panel *B* shows population extinction and nest mortality rates over the study period. Panel *C* shows survey timing; overwinter nest mortality and extinction was measured from September and March surveys and includes the species’ diapause period. Annual extinction was measured from September surveys and includes both diapause and dispersal periods. Exclamation marks in (*C*) indicate developmental stages most strongly impacted by inbreeding depression in experimental studies; Nieminen et al. (35) found reduced survival of inbred larvae during diapause and Saccheri et al. (18) and Haikola (36, 37) reported up to 50% reduction in egg hatching rate of clutches from inbred parents.

Here, we combine genetic and fitness data from the Glanville fritillary butterfly (*Melitaea cinxia*), a classical metapopulation in Åland islands in southwest Finland (33), to test the conditions under which genome-wide genetic diversity predicts fitness and extinction in the wild. The metapopulation comprises over 4,000 suitable patches. These are defined by the presence of the butterfly’s host plants, spread over an area of 50 × 70 km, of which the butterfly occupies about 300 to 500 habitat patches in a given year, with frequent local extinctions and recolonizations (34) (Fig. 1). The high degree of population turnover combined with the collection of genetic and fitness data from many years, thousands of individuals, and hundreds of local populations (habitat patches) makes this an ideal system to ask: i) Is there an association between genetic diversity, extinction risk, and nest mortality across environments and years? And ii) under which conditions are associations strongest? We first quantify the relative effect of heterozygosity versus different population-level environmental and demographic variables on extinction risk and nest mortality in additive models before we explore interactions between heterozygosity and population size/trends and year and their effect on population extinction risk and nest mortality.

Our work builds upon an earlier, now classic study, by Saccheri et al. (18) who found that population extinction risk correlated with heterozygosity levels in the Glanville fritillary metapopulation. However, estimates of genetic diversity in that study were based on only eight markers (seven allozymes and one SSR) and analyses captured only seven local extinctions. The continued long-term monitoring of this population and development of sequencing technology now allows us to examine whether these results are supported with significantly more genetic markers as well as substantially more demographic and environmental data, capturing 276 local extinctions across 6 y. With frequent extinctions and recolonization, the metapopulation is in constant flux (38, 39). High turnover (most patches are continuously occupied for only 1 to 2 y) provides frequent opportunities for recolonization of local populations, often by a single mated female, resulting in the rapid build-up of genetic differentiation (40) and potential for full-sib mating in the next generation (41). The unique combination of large amounts of demographic data on population extinction risk with nest mortality, environmental data, and genome-wide measures of genetic diversity allows an opportunity to gain insights into the relationship between genetic diversity and population extinction risk in a contemporary natural population.

## Results

### Annual Population Extinction Risk.

**Fig. 2.**
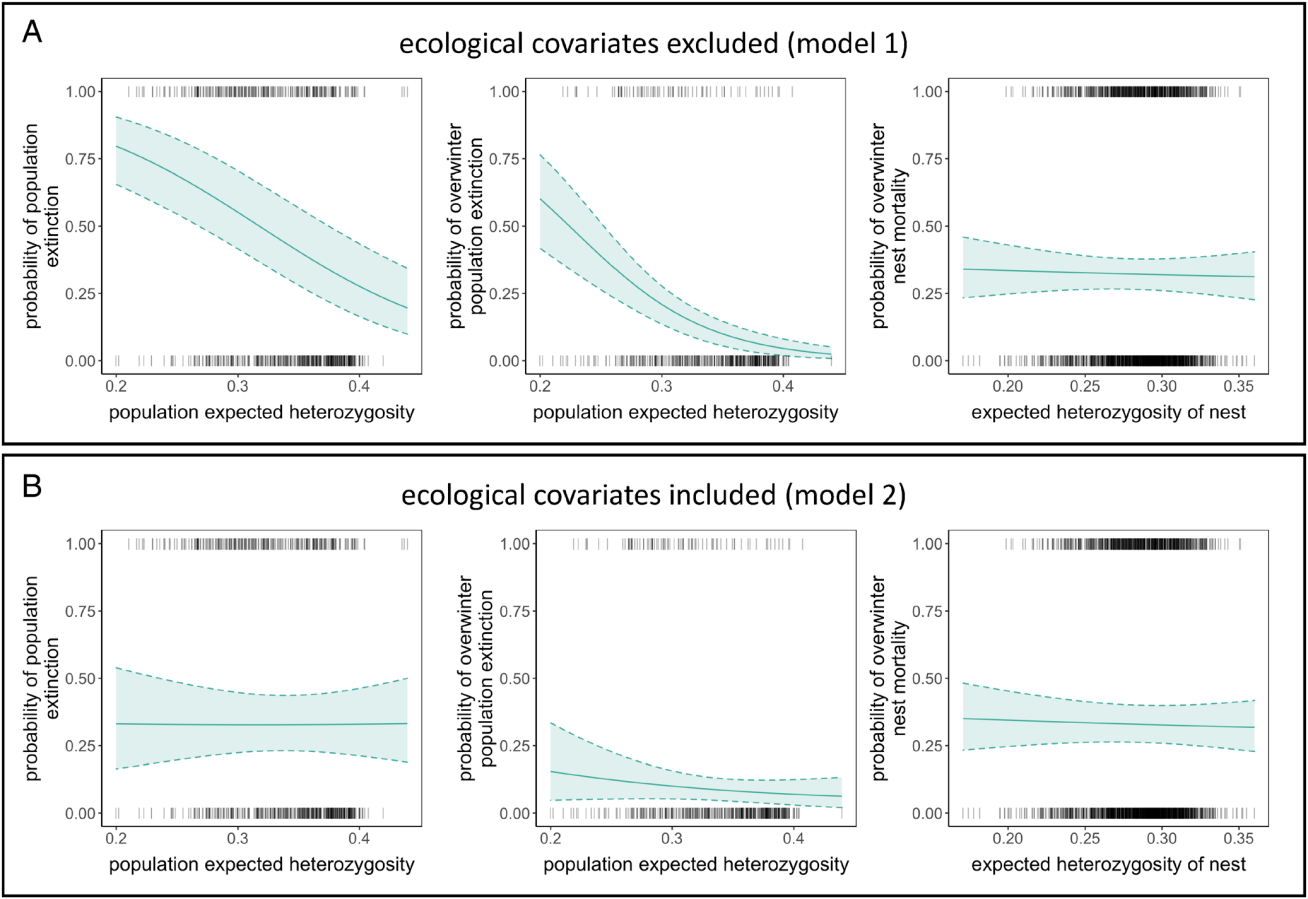
Both annual population extinction and overwinter population extinction decreased with increasing expected heterozygosity in models excluding demographic and environmental variables (*A*). When demographic and environmental variables were included in models this association was not detected (*B*). There was no association between nest mortality and heterozygosity in models excluding (*A*) and including demographic and environmental variables (*B*). Solid lines are posterior mean estimates and dashed lines are 95% credible intervals for model 1 for *A*, and model 2 for *B* from *SI Appendix*, Tables S1, S4, and S7.

Local population extinction measured from annual fall surveys (September to September) showed a strong negative association with expected heterozygosity when it was included as the sole independent variable in a generalized linear mixed model (model 1, Table 1; *SI Appendix*, Table S1; Fig. 2*A*). However, when demographic and environmental variables were added as covariates the relationship between extinction risk and heterozygosity was no longer significant, with credibility intervals overlapping zero (model 2, Table 1; *SI Appendix*, Table S1; Fig. 2*B*). A possible reason for the disappearance of the association between heterozygosity and patch extinction is that heterozygosity is confounded with demographic or environmental variables. For example, associations between demography and population extinction were strong and consistent across all estimated models; populations were more likely to go extinct if they had small local population sizes (nest counts), small patch area, and low connectivity, (*SI Appendix*, Table S1), and population size showed the highest correlation with heterozygosity, at r = 0.6 (*SI Appendix*, Figs. S1 and S2). We used a structural equation model to further partition the independent contribution of heterozygosity to population extinction while controlling for relationships between heterozygosity and other variables. While heterozygosity significantly increased with population size and connectivity (as expected), heterozygosity did not itself have a significant effect on extinction risk (*SI Appendix*, Fig. S3 and Table S2). We further found that higher connectivity, larger patch area, and greater host plant abundance indirectly reduced extinction risk via a positive mediating effect on population size. A global goodness of fit test suggested the structural equation model fit our data well (χ^2^ = 6.0, *P* = 0.53, df = 7)^.^

**Table 1.** Summary of effects of expected heterozygosity (H_e_) on extinction and nest mortality across models including different covariates

			Response variable		
Model no.	Model description	Covariate	Annual population extinction	Overwintering population extinction	Overwintering nest mortality
1	No environmental or demographic variables	H_e_	**−0.55 [−0.75, −0.35]**	**−0.82 [−1.1, −0.55]**	0.02 [−0.13, 0.09]
2	Environmental and demographic variables but no interactions	H_e_	0.002 [−0.25, 0.26]	−0.19 [−0.56, 0.16]	−0.02 [−0.13, 0.09]
3	Interactions with population size and connectivity	H_e_ H_e_: log nest count H_e_: log connectivity	−0.16 [−0.47, 0.15] **−0.32 [−0.63, −0.03]** 0.11 [−0.11, 0.34]	−0.49 [−0.99, 0.03] −0.44 [−1.0, 0.11] 0.12 [−0.21, 0.46]	−0.03 [−0.15, 0.08] −0.02 [−0.15, 0.11] −0.09 [−0.21, 0.04]
4	Interaction with year	H_e_: 2007–08 H_e_: 2008–09 H_e_: 2009–10 H_e_: 2010–11 He: 2011–12 H_e_: 2012–13	−0.30 [−0.80, 0.18] −0.04 [−0.66, 0.57] −0.17 [−0.66, 0.30] 0.17 [−0.37, 0.70] 0.24 [−0.19, 0.67] 0.20 [−0.23, 0.64]	−0.06 [−0.61, 0.51] **−0.77 [−1.5, −0.10]** 0.25 [−0.29, 0.81] −0.54 [−1.1, 0.002]	0.27 [−0.01, 0.56] −0.09 [−0.46, 0.27] −0.19 [−0.39, 0.006] −0.04 [−0.20, 0.12]
5	Interaction with population trend from year t − 1 to t	H_e_: declined H_e_: increased H_e_: stable	0.11 [−0.34, 0.56] 0.09 [−0.52, 0.73] 0.23 [−0.40, 0.90]	−0.63 [−1.4, 0.05] −0.31 [−1.4, 0.88] −0.31 [−1.1, 0.51]	**−0.44 [−0.76, −0.13]** −0.02 [−0.17, 0.13] 0.26 [−0.31, 0.87]
6	Interaction with population trend from year t − 2 to t	H_e_: declined H_e_: increased H_e_: stable	0.12 [−0.59, 0.85] −0.16 [−1.1, 0.82] −0.22 [−0.84, 0.40]	**−1.3 [−3.2, −0.06]** −1.09 [−4.0, 1.2] −0.55 [−2.0, 0.76]	**−0.82 [−1.6, −0.13]** −0.07 [−0.28, 0.13] −0.12 [−0.36, 0.12]

For each of the six models, standardized regression coefficients, and 95% credible intervals are shown. Coefficients for other fixed covariates and random effects are shown in *SI Appendix*, Tables S1, S4, and S7. Credible intervals that do not include zero are bolded.

**Fig. 3.**
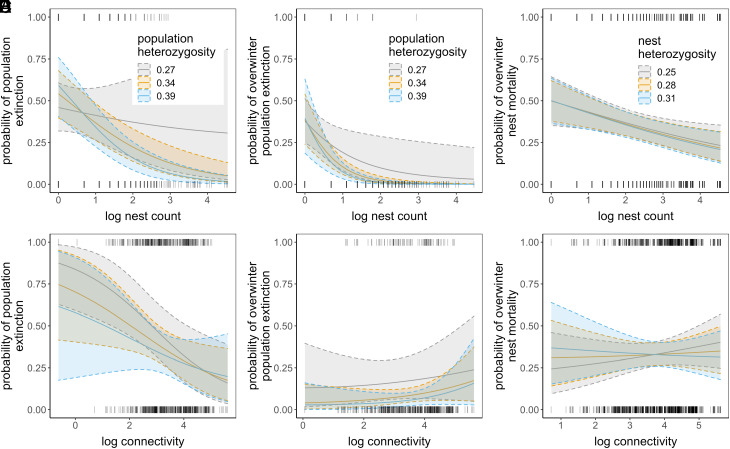
Annual population extinction (*A*), overwintering population extinction (*B*), and nest mortality (*C*) decreased with increasing population size (nest count). Populations with low heterozygosity had an increased risk of extinction compared to high heterozygosity patches only when population size (nest count) was large (*A*). The credible interval of the interaction between expected heterozygosity and population size overlapped zero for both (*B*) and (*C*). Annual population extinction strongly decreased with increasing connectivity (*D*) but no such relationship was found for overwintering population extinction (*E*) nor nest mortality (*F*). All credible intervals for interactions between expected heterozygosity and connectivity overlapped zero. Solid lines are posterior mean estimates and dashed lines are 95% credible intervals from model 3 in *SI Appendix*, Tables S1, S4, and S7.

**Fig. 4.**
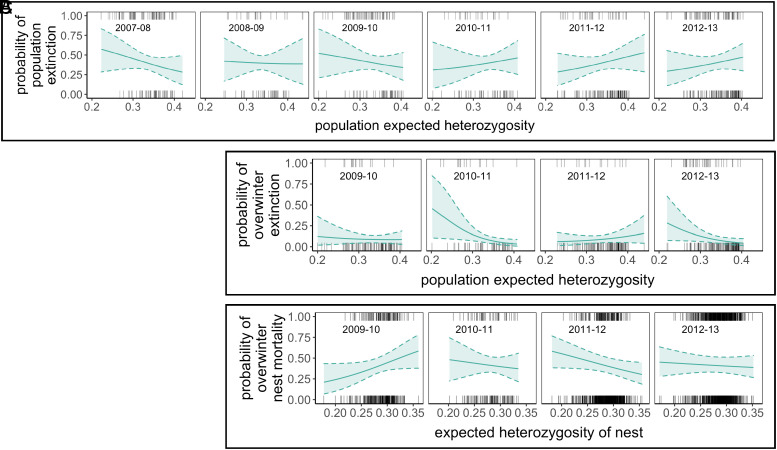
The associations between heterozygosity and annual population extinction (*A*), overwintering population extinction (*B*), and nest mortality (*C*) varied by year. Solid lines are posterior mean estimates and dashed lines are 95% credible intervals from model 4 in *SI Appendix*, Tables S1, S4, and S7.

A model including interactions between heterozygosity and demographic variables revealed that populations with low heterozygosity were more likely to go extinct than high heterozygosity populations in large but not small local populations (model 3, Table 1; *SI Appendix*, Table S1; Fig. 3*A*). Highly isolated local populations tended to go extinct more often when they had low heterozygosity compared to highly isolated populations with high heterozygosity, but credible intervals for this interaction between heterozygosity and patch connectivity overlapped zero (Fig. 3*D*). A model that included an interaction between heterozygosity and sampling year showed that associations between heterozygosity and extinction risk varied in strength and direction. Associations were negative in 2007–2009, and positive in 2010–2012, although credible intervals for all years overlapped zero (model 4, Table 1; *SI Appendix*, Table S1; Fig. 4*A*). Low statistical power likely contributed to high among-year variation in the association between heterozygosity and patch extinction and we explored this using simulations. While there was low power (≤0.27) to detect an effect of heterozygosity on extinction in individual years (Table 2), power was higher (0.74) when considering all years combined.

**Table 2.** Power to detect an effect of expected heterozygosity on annual population extinction and overwintering nest mortality in the Glanville Fritillary butterfly metapopulation

Year	Annual extinction	Nest mortality (*B* = 1)	Nest mortality (*B* = 2.5)	Nest mortality (*B* = 5)
2009	0.22	0.37	0.84	**0.98**
2010	0.13	0.26	0.66	**0.90**
2011	0.20	0.63	**0.99**	**1**
2012	0.27	0.77	**1**	**1**
All years	0.74	**0.98**	**1**	**1**

B is the assumed number of haploid lethal equivalents in the metapopulation. Years with high power (>0.9) are bolded.

**Fig. 5.**
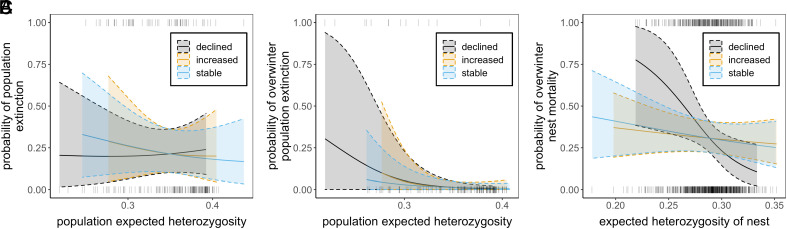
Association between heterozygosity and annual population extinction (*A*), overwintering population extinction (*B*), and overwintering nest mortality (*C*) for local populations that experienced declines, increases, or remained stable over the previous 2-y period. Low heterozygosity nests were more likely to die overwinter when populations experienced declines (*C*). Populations with low heterozygosity were more likely to go extinct overwinter in declining but not stable or increasing populations (*B*), but no such interaction was found when extinction was measured annually (*A*). Solid lines are posterior mean estimates and dashed lines are 95% credible intervals from model 6 in *SI Appendix*, Tables S1, S4, and S7.

Negative impacts of inbreeding may take time to accumulate and require sustained low or declining effective population size (*N*_e_) over multiple generations (42, 43). Given the high turnover of local populations in the metapopulation, current population size does not necessarily reflect demographic history or *N*_e_. Thus, to identify local populations most likely to suffer from inbreeding depression (populations that have declined in size over multiple generations), we separated populations into those that declined, increased, or remained stable over the previous 1 to 2 generations (year t − 1 to t and year t − 2 to t, respectively). Models including interactions between heterozygosity and population trend revealed no association between heterozygosity and population extinction (models 5 to 6, Table 1; *SI Appendix*, Table S1; Fig. 5*A*).

Expected heterozygosity is less sensitive to sample size than observed heterozygosity (44) and it was therefore used as the preferred measure of genetic diversity in our models given the high variance in sample size across populations. However, for comparison, we also ran models using observed heterozygosity as the response variable. Unlike results from models using expected heterozygosity, we found no evidence for an association between observed heterozygosity and population extinction in models excluding or including demographic and environmental covariates (models 1 to 2; *SI Appendix*, Table S3), nor was there any evidence for an interaction between observed heterozygosity and population size. However, observed heterozygosity interacted with patch connectivity to explain population extinction risk; isolated populations with low observed heterozygosity were more likely to go extinct than isolated populations with high heterozygosity, and this association was reversed for highly connected populations (model 3; *SI Appendix*, Table S3 and Fig. S4). Yearly associations between observed heterozygosity and extinction generally followed the same trends as for expected heterozygosity and also had credible intervals overlapping zero (model 4; *SI Appendix*, Table S3). Extinction probability strongly increased with observed heterozygosity in populations that declined or increased over the previous generation (model 5; *SI Appendix*, Table S3) and in populations that remained stable over the previous two generations (model 6; *SI Appendix*, Table S3).

### Overwinter Population Extinction Risk.

Patch extinction measured from annual fall surveys includes the butterfly’s dispersal period, and it is thus possible that a population might go extinct overwinter but is rescued by immigration before the next survey period. For comparison and to minimize the possible confounding influence of dispersal we ran models with overwinter population extinction measured from fall and spring surveys (September to March; Fig. 1*C*). Overwinter extinction showed a strong negative association with expected heterozygosity when it was included as the sole independent variable (model 1; Table 1, *SI Appendix*, Table S4; Fig. 2*A*). However, when demographic and environmental variables were added to the model this association was not detected (model 2; Table 1; *SI Appendix*, Table S4; Fig. 2*B*). Small populations had a high probability of going extinct overwinter compared to large populations across all models (*SI Appendix*, Table S4). Populations with low heterozygosity were more likely to go extinct overwinter than high heterozygosity populations in large but not small local population sizes, but credible intervals for the interaction between heterozygosity and population size overlapped zero (model 3, Table 1; *SI Appendix*, Table S4; Fig. 3*B*). We found no evidence that an interaction between heterozygosity and patch connectivity influenced overwinter patch extinction (Fig. 3*E*). Associations between patch heterozygosity and overwinter patch extinction were negative in 2009–10, 2010–11, and 2012–13, and positive in 2011–12 (model 4, Table 1; *SI Appendix*, Table S4; Fig. 4*B*) but credible intervals overlapped zero for all years except 2010–11. Both models including interactions with population trend showed indications of overfitting (*SI Appendix*, Fig. S5). We therefore reran models excluding covariates with low effect sizes, which revealed the same effect of heterozygosity in declining populations although credible intervals became wider (*SI Appendix*, Tables S4 and S5). Overwinter extinction showed a strong negative association with heterozygosity in populations that had declined over the previous two generations, but not in stable or increasing populations (model 5, Table 1; Fig. 5*B*). The same trend was found when declines were quantified over just a single generation, but credible intervals for the interaction between heterozygosity and population trend overlapped zero (Table 1). No models using observed heterozygosity as a response variable showed evidence for an association between observed heterozygosity and overwintering extinction (*SI Appendix*, Table S6).

### Overwinter Nest Mortality.

To examine potential causes of the increased risk of population extinction in populations with low heterozygosity, we examined whether less heterozygous nests had higher overwinter mortality. We found however no evidence for a main effect of heterozygosity on nest mortality when environmental and demographic variables were excluded (model 1, Table 1; *SI Appendix*, Table S7; Fig. 2*A*) or included (model 2, Table 1; *SI Appendix*, Table S7; Fig. 2*B*) in models. Effect sizes of heterozygosity were weak with wide credible intervals that overlapped zero in these models. In general, demographic variables more strongly influenced nest mortality than heterozygosity. For example, individual nests were more likely to go extinct if they were in patches with small population size and high connectivity (*SI Appendix*, Table S7). We found no evidence for an interactive effect of heterozygosity and local population size (Fig. 3*C*) or patch connectivity (Fig. 3*F*) on nest mortality (model 3, Table 1). Low heterozygosity nests and high heterozygosity nests in small populations had similar overwinter mortality (Fig. 3*D*). Associations between heterozygosity and nest mortality were positive in 2009–10, and negative in the remaining years although credible intervals for all years overlapped zero (model 4, Table 1; *SI Appendix*, Table S7; Fig. 4*C*). Nest mortality strongly increased with decreasing nest heterozygosity in populations that had experienced population declines over 1-2 generations but not in populations that were stable or increasing (models 5 and 6, Table 1; *SI Appendix*, Table S7). This negative association between heterozygosity and nest mortality was stronger in patches that experienced declines over two generations (Fig. 5*C*).

We used simulations to evaluate the power to detect associations between heterozygosity and overwinter survival, assuming different numbers of haploid lethal equivalents (B). Power to detect associations between heterozygosity and overwinter nest mortality was 0.98 with *B* = 1, and 0.99 for *B* = 2.5 and *B* = 5 when considering data from all years combined (Table 2), thus the absence of an association between heterozygosity and overwinter nest mortality across all years is unlikely to be due to low statistical power unless inbreeding depression was exceptionally weak. However, when estimating power to detect associations between heterozygosity and overwinter mortality for individual years, power was high only when assuming *B* = 2.5 to 5 (Table 2) and variation in statistical power among years likely contributed to the variable estimated year-specific associations between heterozygosity and overwinter mortality. The number of lethal equivalents calculated using empirical data from the year where we found the strongest negative association between heterozygosity and mortality (2011–12, Fig. 4*C*) was estimated to be *B* = 1.04.

Expected and observed heterozygosity were highly correlated for nests (Pearson r = 0.82, *SI Appendix*, Fig. S6) and we therefore expect results to be similar regardless of heterozygosity metric used and did not run models with observed heterozygosity.

## Discussion

Here, we take advantage of extensive demographic, environmental, and genetic data from the well-characterized Glanville fritillary butterfly metapopulation to address a long-standing question about the importance of genetic diversity on population persistence. We found a strong negative association between heterozygosity and population extinction when heterozygosity was the sole independent variable included in models. However, this association was not detected when models included demographic and environmental covariates, suggesting a confounding effect between demography and genetics. Including interactions in models revealed that associations between demography and mortality and extinction were generally strong and consistent, whereas associations between extinction and genetic diversity (i.e., heterozygosity) were only strong or detectable under some conditions. For example, while heterozygosity did not predict extinction risk of all currently small populations, it did show a strong negative association with mortality and extinction in local populations with a history of decline. Other associations between genetic diversity and extinction were detectable only in certain years. Our results highlight that studies that do not account for potential confounding factors or inbreeding-by-environment interactions may give misleading inferences about the importance of genetic diversity on population extinction risk.

Although we found a strong negative association between heterozygosity and extinction when heterozygosity was included as the sole independent variable in models (Fig. 2*A*), this association disappeared when demographic and environmental covariates were included (Fig. 2*B*). This suggests that heterozygosity is confounded and a failure to account for demography and environment may lead to false positive (or negative) associations between genetic diversity and extinction risk (45). Because reductions in population size cause concomitant reductions in genetic diversity, the two variables are often highly correlated and difficult to disentangle in nature (46, 47). In our data, population size and expected heterozygosity of populations were moderately and positively correlated (Pearson r = 0.6, variance inflation factors <2). While this level of collinearity is below the threshold known to cause problems for parameter estimation in regression models (48), the association between the two variables is nonlinear (*SI Appendix*, Fig. S2), and typical metrics might thus underestimate their level of confounding. The inclusion of interactions between population size and heterozygosity allowed some level of stratification to help alleviate problems with multicollinearity; this revealed that heterozygosity predicted extinction risk in large but not small populations (Fig. 3). We thus conclude that associations between heterozygosity and extinction are not consistent across populations, and the strong general association detected between them in our first model was largely driven by a confounding effect with population size. Ecological factors are often not included in studies of inbreeding (45), and with increasing ease of sequencing, attempts to use genetic variation alone as a proxy of population viability have become more common. Our results underline another cause of concern when interpreting broad-stroke associations between genetic variation and conservation status: It is likely that confounding demographic effects may underlie some conflicting results that have previously demonstrated either weak (49) or strong (50) correlations between genetic diversity and IUCN Red List rank.

The inclusion of interactions with demographic variables in our models revealed that associations between heterozygosity and extinction were important or detectable in certain contexts and environments. We expected to find stronger impacts of heterozygosity on extinction in small versus large populations, due to increased drift load in small populations (11) and universally low probability of extinction for those that are large. In contrast to this expectation, low genetic diversity did not contribute to elevated mortality and extinction in small populations beyond demographic effects. There can be several reasons for this. Demographic stochasticity is expected to be stronger in small versus large populations (51–53), and strong Allee effects might drive small populations to extinction before negative impacts of inbreeding have had time to accumulate (47). The latter is not broadly supported in the literature (50), and while Allee effects are present for *M. cinxia* in Åland (51), experimental work suggests that inbreeding likely contributes to observed Allee effects by reducing mating success in small populations after 1 to 2 generations of sib-mating (36). Alternatively, the lack of an association between heterozygosity and extinction in small populations might be expected if census population size does not reflect effective population size or recent demographic history. Negative fitness effects of inbreeding may require sustained bottlenecks to accumulate (54). This scenario was recently exemplified by Wootten et al. (55) who found higher genetic estimates of inbreeding in mountain goats that had a stable current population, but suffered a large historical bottleneck compared to caribou that have declined only recently. The dynamic nature of the Glanville fritillary metapopulation means that local population trajectories are highly variable and that currently small populations may not have remained small for long periods (*SI Appendix*, Fig. S7). When interactions with population history were included in our models, we found that overwinter population extinction and nest mortality strongly declined with increasing expected heterozygosity in populations that declined over the previous 1 to 2 generations, with no such associations found in stable or increasing populations (Fig. 5 *B* and *C*). Interestingly, we did not find this same pattern when extinction was quantified over the entire year (September to September), but this is likely due to the presence of the rescue effect; we found several instances where declined populations that went extinct overwinter were recolonized before the next fall survey. Another explanation for a lack of association between heterozygosity and extinction in small populations might be the presence of purging. This is supported by our finding that small but not large populations have a higher number of heterozygotes than expected (i.e. observed heterozygosity is greater than expected heterozygosity, *SI Appendix*, Fig. S6). This pattern is consistent with strong directional selection against homozygotes, but might alternatively be explained by metapopulation dynamics. For example, small populations are often established via colonization by two or more unrelated females (41), which can lead to an excess of heterozygotes in the absence of purging. We find this to be the more likely scenario given that previous studies have found substantial genetic load in small, isolated populations in Åland (36). Alternatively, this result might be explained by a sampling effect, where different allele frequencies in males and females result in an excess of heterozygotes in offspring (56).

Comparisons of extinction periods including and excluding dispersal, and the inclusion of interactions between heterozygosity and patch connectivity allowed us to further test how dispersal and genetic rescue might confound associations between genetic diversity and extinction risk. Immigration is expected to be lowest when populations are highly isolated, and we thus expected to find stronger impacts of heterozygosity on extinction in isolated compared to connected populations. We found some evidence for this—isolated populations with low heterozygosity tended to go extinct more often than isolated populations with high heterozygosity (Fig. 3*D*). Although uncertainty was high for the interaction between connectivity and expected heterozygosity, which had wide credible intervals overlapping zero, the effect of an interaction between connectivity and observed heterozygosity on population extinction was significant (*SI Appendix*, Fig. S4 and Table S3). These results are consistent with those of Saccheri et al. (18) who found that associations between heterozygosity and extinction risk were most pronounced in small, isolated populations in the same metapopulation more than a decade prior to the initiation of our study. However, we found no evidence of this when extinction was quantified overwinter, for which main effects of connectivity were also absent or even slightly positive (Fig. 3 *E* and *F*). This further highlights the importance of the rescue effect. There is no chance for rescue in either isolated or connected populations overwinter and thus low heterozygosity populations may have an elevated risk of extinction regardless of connectivity (e.g., Fig. 3*E*). Alternatively, we may see stronger associations for extinction quantified annually as it captures negative fitness effects of inbreeding that have accumulated over the entire life cycle of the butterfly and that may spillover into the next generation (Fig. 1*C*). For example, inbred larvae that happen to survive the winter are more likely to go on to mate with full sibs in isolated populations, whose clutches will then suffer from strongly reduced egg hatching rate (18, 35). A further difficulty is that our measure of population connectivity may not sufficiently capture the complex patterns of dispersal and gene flow that we have recently demonstrated in this system (40, 57), which may have contributed to uncertainty in relationships for expected heterozygosity. Overall, these results highlight that dispersal helps to alleviate the elevated extinction risk associated with reduced genetic diversity. Many natural populations are spatially structured and linked by dispersal, yet studies of inbreeding often treat populations as completely isolated (45, 58, 59). This can lead to an absence of association between genetic diversity and extinction even when bottlenecks are severe and inbreeding effects on fitness are large (54, 60).

In addition to the estimated low power to detect inbreeding effects in individual years (Table 2), inbreeding-by-environment interactions might also explain why we see associations between heterozygosity and extinction and mortality only in certain years and conditions. Several studies have reported stronger importance of heterozygosity on fitness in harsher environments, and a complete lack of negative fitness effects despite strong inbreeding in benign environmental conditions (25–27). Population growth rates in the butterfly metapopulation are closely tied to weather conditions—especially water balances during spring and summer months (61, 62), and quality of habitat varies strongly across years, patches, and among microhabitats within patches (63, 64). During our study period, the summers between the 2007–08 and 2009–10 were characterized by low vegetation productivity (62), low overall metapopulation size, and high extinction rates (Fig. 1*B*), possibly reflecting relatively harsh conditions for adults and larvae. Our results support this since negative associations between heterozygosity and population extinction were strongest during these periods (Fig. 4*A*). On the other hand, overwintering extinction and nest mortality are probably governed by the harshness of conditions during winter, for which we know less about in this system but might be related to variability of insulating snowpack (65). We have previously found that within-population variation in nest mortality risk is partially explained by variation in microhabitat quality (63, 64). This microhabitat variation may explain why individual nest mortality does not always translate to patch extinction.

### Caveats.

An advantage of our study system is that we can assess genetic contribution to mortality and extinction risk over hundreds of local populations that differ in ecological context. However, local populations are connected by gene flow and do not represent independent genetically distinct populations. Thus, the extinction of a local population does not mean the loss of a genetic lineage. While the loss of very small and ephemeral local populations may not impact larger population dynamics, our results suggest that the negative impacts of low genetic diversity extend to large populations and other ecological contexts that are independent of population size. Future work could test the importance of genetic variation to larger-scale metapopulation dynamics that are realized over many connected patches. For example, using spatially explicit simulations, Nonaka et al. (32) showed that inbreeding is more common and local metapopulation extinction risk is higher in smaller patch networks than larger networks. Semi-independent networks do go extinct in this system, but with much less regularity than individual patches (66). Thus, to test the impact of genetic variation on network-level extinction, future work would need many more years of genetic data and a wider spatial scope. The question of how inbreeding scales up to network extinction risk is timely given that increasing spatial synchrony in weather conditions across Åland puts the entire metapopulation at risk of decline under extreme weather events (61, 62).

Additionally, despite the large sample sizes deriving from genotyping thousands of larvae and over 6 y of intensive fieldwork to collect demographic and environmental data, we still had relatively low statistical power to detect associations between genetic diversity and extinction risk and mortality within years. We therefore cannot exclude the possibility that there could be year-specific effects of inbreeding on extinction and nest mortality we did not have the power to detect.

## Conclusions

The ever-increasing access to genomes of species of conservation concern, or even extinct species, presents unique opportunities to understand the role that genetic diversity has on population viability. By combining genetic data with demographic and environmental information at a large scale, our study highlights the many challenges involved in trying to disentangle the effect that demographic, environmental, and genetic variations have on fitness and extinction risk in natural populations. While we found a strong negative association between heterozygosity and extinction when ecological variables were excluded from models, we demonstrate that this association was likely driven by a confounding effect of heterozygosity with local population size. Controlling for confounding factors by including interactions between heterozygosity and demographic variables and comparing extinction periods including and excluding dispersal did reveal associations between heterozygosity and extinction in certain contexts. We thus conclude that low genetic diversity can be an important predictor of extinction risk when confounding influences of demography and dispersal are controlled for. Had we failed to include demographic and environmental variables in our models of extinction risk, we would have concluded that effects of low genetic diversity were strong and consistent across populations and environments. From such a conclusion it would be tempting to use genetic diversity alone as a proxy of population viability, which in this case would lead to poor prioritization of conservation effort. On the other hand, had we considered demographic and environmental variables in our models but failed to include their interactions with genetic diversity, we would have erroneously concluded that genome-wide genetic diversity was unimportant for explaining extinction risk. Our results thus highlight that strong inferences about the importance of genetic diversity for predicting fitness and extinction of natural populations require accounting for the effects of demographic and environmental factors.

## Materials and Methods

### Study System.

Here, we use long-term monitoring survey data of the Glanville fritillary butterfly (*M. cinxia*) in the Åland Islands, SW Finland (see ref. 34 for details). In Åland the butterfly’s habitat consists of around 4,000 meadows and pastures (hereinafter “patches”) containing one or both of the host plant species, *Plantago lanceolata* and *Veronica spicata*. The Glanville fritillary is univoltine in its northern range, with mating occurring in the butterfly’s natal patch in early summer. Females are mostly monogamous (67) and can lay several clutches of 50 to 250 eggs (68) on host plants either in the female’s natal patch, in a new patch(es) following dispersal, or both. Eggs develop into gregarious larvae, which build communal nests to diapause overwinter, from approximately September to late March (Fig. 1*C*). The metapopulation is highly dynamic, and in a given year only around 20% of available patches are occupied. Local populations are small (e.g., population size in our data ranged from 1 to 94 nests with a median population size of three nests) and few persist more than a couple of years (e.g., median period of continuous patch occupancy in our data is 2 y). Thus, local extinctions are frequent and are balanced by recolonization of unoccupied patches by dispersing mated females.

Communal winter nests are conspicuous and found at the base of the host plants, and mainly consist of full-sibs (50 to 250 larvae), although merging and splitting of nests can occur (69). Every year since 1993, each of the 4,000 patches have been visited in September to systematically mark and count larval nests. Patches that were found to be occupied in September are then revisited in March to document overwinter nest survival. Three decades of research using these survey data have been used to characterize the ecological and evolutionary determinants of metapopulation dynamics in this system. Mortality during the larval stage is high, and larval survival is correlated within nests (68). Individual nests die for many reasons, including small group size (68), and poor host plant or microhabitat quality (64). Extinctions of local populations are mainly explained by small population size, small patch size, low patch connectivity, and to a lesser extent poor habitat quality (e.g. high percentage of cattle grazing) (70, 71). Broad-scale climate and weather conditions further explain metapopulation growth rates at large spatial scales, with aridity during the late spring and early summer being especially important (61, 62). Reduced fitness has been documented in several traits across the life cycle of the butterfly after 1 to 2 generations of full-sib mating, which further contributes to mortality and extinction. The most severe fitness effects of inbreeding appear to manifest during reproduction, where inbred parents have severely reduced egg hatching rate, and winter diapause, where inbred larvae are smaller, build poorer quality nests, and have reduced survival compared to cross-bred larvae (18, 35).

### Sampling and Genomic Data.

For the years 2007–2012, 10,000 larvae (three per nest) in all occupied patches in a 10 × 10 km region on mainland Åland (Saltvik), and two islands (Föglö and Sottunga), were genotyped to represent a broad temporal sample (Fig. 1*A*). The three sites differ in metapopulation size and amount and configuration of available habitat (66). They likely are not connected by contemporary migration as butterflies do not readily disperse across water, but Sottunga and Saltvik share a recent demographic history as butterflies on Sottunga derive from 72 founders from the mainland of Åland, which were intentionally introduced by researchers in 1991 (72). Results from analyses analyzing Saltvik alone (*SI Appendix*, Tables S8–S10) or all three sites combined were similar, thus we focus on the combined analyses here. Samples were genotyped at 245 SNPs that included 36 introns, 165 SNPs that showed expression differences in experimental flight treatments, and 38 gap-filling SNPs that were selected to even out the distribution of loci across chromosomes. These loci are well distributed across the genome (*SI Appendix*, Table S11). Loci were developed as part of a larger panel for a previous study, and represent the subset of loci found to be unlinked in pairwise tests of linkage disequilibrium in Åland that had SNP call rates >95% (73). Further details can be found in (69, 73). After excluding individuals with failed DNA extraction and genotype call rates of <95%, 8,322 individuals remained. We calculated observed and expected heterozygosity of both nests and populations using the R package hierfstat v0.5-11 (74). Heterozygosity was measured at the nest- instead of individual-level as calculating over multiple individuals gives better estimates of heterozygosity (44) and mortality data was available for entire nests only. Expected heterozygosity is less sensitive to sample size than observed heterozygosity (44) and it was therefore used as the preferred measure of genetic diversity for our models given the high variance in sample size across populations and nests. Observed and expected heterozygosity were highly correlated for nests (r = 0.82; *SI Appendix*, Fig. S6) but not populations (r = 0.44; *SI Appendix*, Fig. S6). Thus, we explored the impact of heterozygosity measure on model outcomes by comparing population extinction models parameterized with each. Heterozygosity values calculated using just introns, potentially adaptive, or gap-filling SNPs were highly correlated with heterozygosity calculated using all SNPs (*SI Appendix*, Fig. S8), suggesting that our selection of loci represents genome-wide genetic diversity well.

### Does Heterozygosity Predict Population Extinction and Nest Mortality?

Our main objective was to test whether and under which conditions heterozygosity contributes to population extinction risk and overwinter nest mortality. Annual extinction was assessed from nest counts obtained during the autumn surveys from 2007 to 2012 (Fig. 1*C*). If a previously occupied patch was found to be unoccupied (i.e., no nests were found) in the following autumn, it was assumed to have gone extinct (34). However, there is a period of dispersal in between autumn surveys (Fig. 1*C*), which means that a patch that goes extinct overwinter can be rescued before the next survey period, or apparent extinction can occur if all female butterflies leave a patch during summer without laying any clutches. To control for this confounding rescue by dispersal, we additionally quantified overwintering population extinction by comparing nest counts from autumn to the following spring during which there is no dispersal (Fig. 1*C*). Overwinter mortality of individual nests was assessed by revisiting all nests found during the autumn survey in the following spring. Digitized data from spring surveys were available only from 2009 onward, and thus our analysis of overwintering extinction and nest mortality span 4 y from 2009 to 2012. The data underwent several filtering steps. We removed individuals with less than two genotypes per nest or population as this was the minimum sample required to calculate expected heterozygosity. After removing populations with missing autumn survey data, the final sample size for yearly population extinction models was n = 643 (268 extinction events), spanning 279 unique populations and 7,501 contributing genotypes. Overwintering population extinction models had a final sample size of n = 460, spanning 247 unique populations, and 5,797 contributing genotypes. For nest mortality models only, we removed nests that contained larvae from more than one full-sib family (i.e. merged nests) using a previously published genetic reconstruction of sibship (69). The final sample size for overwinter nest mortality models was n = 1,742 nests (638 mortality events), spanning 237 unique populations and 4,636 contributing genotypes. The rate of annual population extinction ranged from 0.26 to 0.57 per year, overwintering population extinction from 0.16 to 0.26, and overwintering mortality of nests from 0.22 to 0.48 (Fig. 1*B*). Patch age (i.e., period of continuous occupancy) ranged from 1 to 20 y, with a median of 2 y. The maximum age of a patch did not strongly differ among those patches that persisted versus those that went extinct during our study period (*SI Appendix*, Fig. S9).

Population extinction, overwintering population extinction, and overwintering nest mortality measured from year t to t + 1 were used as response variables in generalized linear mixed models with binomial error distribution. Models were implemented in R-INLA (75) and included a spatial random effect to account for the nonindependence of metapopulation processes occurring in nearby patches. Briefly, INLA provides an alternative to MCMC for fitting latent Gaussian models (i.e., hierarchical models where there are unobserved normally distributed random variables) that account for spatial dependency in observations by incorporating Gaussian random fields (GRFs). GRFs reflect a spatially continuous random process where observations are correlated in space, and continuous space is approximated using a “mesh” that is initially defined by spatial locations of sampling points. Spatial covariance in the spatial random effect was included as a Matern function. Temporal autocorrelation was accounted for by including a temporal random effect with a first-order autocorrelation term. Marginal variance, *σ*^2^, the spatial range of spatial autocorrelation, and temporal autocorrelation (*ρ*) were estimated from the model.

As many ecological factors can potentially contribute to variation in population size and turnover (71), we measured several ecological parameters for each population and included them as covariates in our models. During the autumn surveys, patch quality was assessed through four measures: the amount of dominant (based on total coverage estimates of the two host plant species) host plant on a scale of 1 to 3 (with a score of 1 indicating sparse host, and 3 indicating at least one large group of hosts that could support >10 larval groups; i.e. host plant abundance), the proportion of dry host plants (based on phenotypic symptoms of drought stress such as brown or wilted leaves), the proportion of host plants growing in low vegetation [females typically lay eggs in low vegetation that provide the required warmer microhabitats for the larvae (64)]; and the proportion of the patch that was grazed (more details in ref. 34). We also calculated metapopulation variables that are known to be important for population dynamics (71). Patch connectivity was calculated using the incidence function model (76):

[1] $$S_{i}=\sum_{i\neq j}\exp(-\alpha d_{\mathrm{ij}})N_{j},$$

where $d_{\mathrm{ij}}$ is the distance between focal patch *i* and source patch *j*, $N_{j}$ is the population size (number of larval nests) of source patch *j* in the previous year, and α is a constant scaling assumed to be equal to 1/mean dispersal distance of the species, which was set to 1 km based on previous work (76). Source patch *j* included any patch that was occupied in the previous year, including populations that were not genotyped for this study. Dispersal is highly complex in this species and depends on habitat quality, weather conditions, individual genotypes, and their interactions (40, 57). It would be difficult to capture these complexities in a single metric and we thus chose one that could feasibly be used in other studies without such detailed information. The metric of structural connectivity used here may not perfectly capture patterns of dispersal, but its high correlation with patch occupancy, population growth rates, and extinction (38, 66, 71) suggests that general patterns are accurately reflected. For each patch, we additionally calculated ${\mathrm{Ntrend}}_{i}$, describing the growth rate trend in the vicinity of patch *i* from time t − 1 to t:

[2] $${\mathrm{Ntrend}}_{i}=S_{i,t}-S_{i,t-1}.$$

${\mathrm{Ntrend}}_{i}$ captures growth rate at large spatial scales and tended to be either all positive or all negative for a given year, reflecting metapopulation growth rate. We included the log of population size in year t, ${\mathrm{Ntrend}}_{i}$, the log of patch area, the log of connectivity, host plant abundance, the proportion of host growing in low vegetation, the proportion of dry host plants, and the proportion of the patch that is grazed in the models described below.

To understand the conditions under which the impacts of genetic diversity on extinction and mortality are strongest, we estimated six different statistical models for both the population and nest-level data. Unless otherwise indicated, all models contained spatial and temporal random effects, and a random effect of population identity. The first model included only heterozygosity as a fixed effect, to reflect the typical case where ecological and demographic variables are not accounted for. The second model tested the impact of including relevant demographic and environmental variables, and included all covariates described above with no interactions. The third model additionally included interactions of heterozygosity with both population size and patch connectivity, as we expected the effects of heterozygosity to be most important in small, isolated populations as large and well-connected populations may have low probability of extinction regardless of heterozygosity. The fourth model included an interaction between heterozygosity and year, as the effects of heterozygosity might be more pronounced in years with, e.g., unfavorable or more stressful weather conditions (77, 78). While weather conditions across different life stages impact population growth rates (61), high aridity during early spring and summer is especially associated with decreased population growth (62). Summers between 2007–08 and 2009–10 were characterized by high aridity and we thus expect associations between heterozygosity and extinction to be strongest in these years. Year was included as a categorical variable because extinction rates are negatively autocorrelated in time, and we found no evidence for temporal trends in extinction (Fig. 1*B*). The temporal random effect was excluded from this model. Finally, we tested for differential impacts of heterozygosity on population extinction and nest mortality for populations that declined, increased, or remained stable in the number of nests over the previous 1 (model five) or 2 y (model six), as the negative impacts of inbreeding may take time to accumulate (36, 37). In addition to the above covariates, these models included an interaction of categorical population trend and heterozygosity. For model five including an interaction with patch growth trend from time t − 1 to t, we excluded patches that were unoccupied in time t − 1. Final sample sizes for model five were n = 344 and n = 236 for annual and overwintering population extinction, respectively, and n = 1,251 nests for nest mortality. For model six including an interaction with population trend from time t − 2 to t, we further excluded patches that were unoccupied in time t − 2, and categorized populations as declined only if they declined from both time t − 2 to t − 1 and t − 1 to t and increased if they increased during both periods. If they neither declined nor increased during both periods they were categorized as stable, which in this context means they either remained the same or fluctuated with no directionality from time t − 2 to t (*SI Appendix*, Fig. S7). Final sample sizes for model five were n = 212 and n = 132 for annual and overwintering population extinction, respectively, and n = 890 nests for nest mortality. All continuous variables were normalized to a mean of zero and SD of one before inclusion in the models to allow comparison of effects among covariates. Choice of priors for random effects and model validation are explained in *SI Appendix*.

Results from our population extinction analysis revealed a potential confounding effect between expected heterozygosity and local population size (nest count). Pairwise Pearson correlations of all covariates were 0.6 (*SI Appendix*, Fig. S1) and variance inflation factors under two, suggesting regression coefficients should be unbiased (48). However, we further used a structural equation model to estimate the independent contributions of population size and heterozygosity to patch extinction while accounting for their direct relationships with habitat quality variables. The model was estimated using piecewiseSEM (79) by specifying three regressions: i) a linear mixed effect model with heterozygosity as the response explained by population size and connectivity, which are expected to increase patch heterozygosity, ii) a linear mixed effect model with population size as the response explained by connectivity and habitat quality variables, and iii) a generalized linear mixed effect model with binomial error with patch extinction as the response and all covariates. Models alternatively fit with a directed relationship between heterozygosity and population size in the other direction (i.e., population size ~ heterozygosity) or including no directed relationship but a correlated error term between the two covariates, produced similar path coefficients but poor fitting models (χ^2^ = 48.9, *P* < 0.001, df = 7; χ^2^ = 45.3, *P* < 0.001, df = 7, respectively).

We estimated *B* (number of haploid lethal equivalents or inbreeding load) for overwinter survival for the year where we found the strongest negative year-specific association between heterozygosity and overwinter mortality (model 4), following the approach of [**2**]. First, we convert the estimate of heterozygosity for each nest ($H_{\mathrm{SNP}}$) to a heterozygosity-based measure of inbreeding for that nest as

[3] $$F_{h}=(H_{SNP,0}-H_{\mathrm{SNP}})/H_{SNP,0},$$

where $H_{SNP,0}$ is the highest observed value of expected heterozygosity among all nests in the metapopulation (80, 81). $F_{h}$ therefore measures the proportional reduction in heterozygosity relative to the most heterozygous nest, which is assumed here to be noninbred. We then calculated *B* as

[4] $$B=-\text{log}\left(\frac{S_{F_{h,max}}}{S_{F_{h,0}}}\right)/F_{h,max},$$

where $S_{F_{h,max}}$ and $S_{F_{h,0}}$ are the overwinter survival probabilities for nests with the highest and lowest inbreeding ($F_{h}$), respectively.

### Power Analysis.

We used simulations to evaluate the power to detect effects of heterozygosity on overwinter survival and local patch extinction. We model overwinter nest-level mortality as a function of the inbreeding coefficient *F* of a nest. The assumed probability of a nest surviving over winter was $S_{\mathrm{fam}}=e^{-BFS_{0}}$ where *B* is the assumed number of haploid lethal equivalents in the metapopulation, and *S*_0_ is the probability of overwinter survival for nests with the highest heterozygosity, which are assumed to be noninbred (*F* = 0). We set *S*_0_ to 0.7 (our empirical estimate of the overwinter survival probability for nests with the highest heterozygosity in 2011, Fig. 4*C*) and then ran separate power analyses assuming *B* = 1, 2.5, or 5, to span a reasonable range of inbreeding loads for survival in nonmodel species (82).

We assumed in our power analysis that probability of population extinction was a logistic function of heterozygosity. We evaluated the power to detect the most extreme year-specific effect of heterozygosity (*H*) on extinction probability observed in our empirical analysis to determine whether our data provided sufficient power to detect relatively large effects of heterozygosity on extinction. Therefore, the true population extinction probability (P(Ext)) in these simulations was assumed to be 0.6 for populations with the lowest *H*, and 0.25 for those with the highest *H*, consistent with the estimated effect in 2007 (Fig. 4*A*). Based on this assumption, a linear model of the log odds of extinction versus *H* is log(P(Ext)/(1 − P(Ext)) = L = 1.58−6.14H. Whether a particular population with a given value of *H* went extinct in the simulations was determined by binomial sampling with probability = P(Extinction|*H*) = 1/(1 + e − L).

We estimated the statistical power to detect effects of heterozygosity on overwinter nest mortality and population extinction for each year separately (accounting for variation in sample size across years, and variation in heterozygosity across populations and families within each year), and when including data from all years combined. We ran 5,000 simulation replicates for each year and for all years combined. For each simulation replicate, we modeled survival or extinction as a function of heterozygosity using logistic regression in R (glm function, with family = binomial). Our power analyses account for sampling error due to finite sample sizes within families and patches, and due to using a finite number of loci to estimate genome-wide heterozygosity. We defined power as the proportion of 5,000 simulation replicates where the response variable was statistically significantly related to heterozygosity in the logistic regression (*P* ≤ 0.05). Power analysis methods details are in *SI Appendix*.

## Supplementary Material

Appendix 01 (PDF)

## Data Availability

Heterozygosity, survey, and ecological data and R scripts needed to reconstruct statistical analyses are deposited in DRYAD at https://doi.org/10.5061/dryad.905qfttrg (83). Previously published data were used for this work (https://doi.org/10.5061/dryad.d461s) (84).
