# Supplementary material for: Demography and environment modulate the effects of genetic diversity on extinction risk in a butterfly metapopulation
Source: Proc Natl Acad Sci U S A. 2024 Aug 8;121(33):e2309455121. doi: 10.1073/pnas.2309455121 (PMC11331070; doi:10.1073/pnas.2309455121)
Supplement: Supplementary file 1 — Appendix 01 (PDF) [file pnas.2309455121.sapp.pdf]

PNAS

Supporting Information for
Demography and environment modulate the effects of genetic diversity on extinction risk in a butterfly metapopulation

Michelle F. DiLeo, Abhilash Nair, Marty Kardos, Arild Husby, Marjo Saastamoinen

Corresponding authors: Michelle F. DiLeo, Marjo Saastamoinen
Email: michelle.dileo@ontario.ca; marjo.saastamoinen@helsinki.fi

This PDF file includes:

Supporting text
Figures S1 to S9
Tables S1 to S11

Supporting Information Text

Supplementary Methods and Results

CHOICE OF PRIORS

Model specification and choice of priors followed those of Schulz et al. (1) who modeled long-term dynamics of occupancy and abundance in the *Melitaea cinxia* metapopulation. We constrained the spatial correlation to stay within the domain of our study system by defining priors of the Matern covariance function as ρ_0=20, α_ρ=0.9, σ_0=1, and α_σ=0.25, where σ^2 is the marginal variance, ρ is the range, and $\Pr(\rho < \rho_0) = \alpha_\rho$ and $\Pr(\sigma > \sigma_0)$. We used a PC prior for the patch random effect to penalize model complexity (2), specified by setting the probability of variance exceeding 1 at 0.25.

MODEL VALIDATION

We investigated the distribution of PIT (probability integral transformation) values as a measure of model adequacy. PIT values are a leave-one-out predictive measure of fit (3), where uniformly distributed values indicate good fit and skewed and u-shaped distributions indicates bias and dispersion, respectively. We applied the adjustment of (4) to account for the binomial outcome of our models. Distributions of PIT values were uniform for most models indicating good fit. Models 5 and 6 for the overwintering population extinction were skewed (Fig. S5). Removing unimportant covariates to avoid overfitting the models improved the distribution of PIT values and did not change the overall outcome of effect of heterozygosity on overwintering patch extinction (Table S5).

POWER ANALYSIS

We sought to evaluate the power to detect effects of heterozygosity on nest survival and population extinction. The power analyses were designed to account for real variation (process variance) in heterozygosity among nests and patches, and for variation associated with error (error variance) arising from imprecision related to finite sample size (limited sampling of offspring within a nest or patch) and a finite number of genotyped loci (limited sampling of each individual's genome).

Accounting for error variance required an estimate of the standard error in estimated heterozygosity for an individual ($\widehat{H}$) and an estimate of the standard deviation in realized heterozygosity (*H*) among siblings within nests. We used simulations of a large inbred family to estimate the standard error (se) of individual $\widehat{H}$ (se($\widehat{H}$)) in *Cinxia*. The simulated genome had 31 chromosomes (5) of equal size, with a single crossover per chromosome per meiosis on average (i.e., each chromosome was 100 centiMorgans). The simulations included genotypes at 245 SNPs (as in our empirical data) that were randomly distributed across the genome. Each SNP had expected heterozygosity equal to the average expected heterozygosity in our empirical data. We simulated Mendelian segregation and recombination in the pedigree of 2,000 offspring of full siblings, such that the pedigree-based inbreeding coefficient *F* was 0.25 in the simulated offspring. We calculated a genomic metric of *F* for each offspring as the proportion of its genome in identical-by-descent chromosome segments (F_{ROH}), which is expected . We estimated se($\widehat{H}$) as the average absolute value of the residuals from a linear regression of $\widehat{H}$ (proportion of heterozygous SNPs) on F_{ROH}.

Next, we estimated the standard deviation in realized heterozygosity among siblings within nests. The standard deviation in estimated heterozygosity among full siblings ($\sigma(\widehat{H})$) within nests has two contributors: variation arising from imprecision associated with finite number of loci (which we measured as se($\widehat{H}$) as described above), and variation in actual individual genome-wide heterozygosity (*H*) due to the stochastic effects of Mendelian segregation and recombination (6), here measured as σ(*H*). We can thus estimate σ(*H*) as σ(*H*) = $\sigma(\widehat{H})$ – se($\widehat{H}$)). We measured $\sigma(\widehat{H})$ as the average within-nest standard deviation in $\widehat{H}$ among all 3,291 nests included in our empirical analyses of patch extinction. We then estimated σ(*H*) as $\sigma(\widehat{H})$ - se($\widehat{H}$) = 0.0272 – 0.018 = 0.0092.

For all of our power analyses we assumed that the raw estimate of average heterozygosity ($\hat{\bar{H}}$) for a given nest was correct. In other words, we assumed that real average heterozygosity within a nest ($\bar{H}$) was equal to $\hat{\bar{H}}$. We therefore model nest survival as a function of $\hat{\bar{H}}$ as described in the main text. Power analyses were run separately for individual years, and then using the data combined across all year.

Power analysis for effects of heterozygosity on nest survival:

1. We began by simulating nest survival as a function of *F* as described in the main text. *F* for each family is determined as 1 – H/H_0 (7), where H_0 is the highest empirical value of $\bar{H}$ among nests in the metapopulation. This is equivalent to assuming the nest with the highest *H* in the metapopulation is non-inbred and that nests with lower heterozygosity are more highly inbred.

2. We then test for a statistical association between simulated estimates of average nest heterozygosity ($\hat{\bar{H}}$) and survival. The number of nests and the number of sampled offspring in the power analyses were identical to those in the empirical analyses. The simulated real heterozygosity for each individual in a nest (*H*) was drawn at random from a normal distribution with mean $\bar{H}$ and standard deviation σ(*H*). The simulated estimate of heterozygosity for each individual ($\hat{H}$) in each nest was drawn from a normal distribution with mean *H* and standard deviation se($\hat{H}$). The simulated value of $\hat{\bar{H}}$ for each nest is calculated as the average $\hat{H}$ among individuals within the nest.

3. For each of 5,000 replicates, we test for a statistical association between $\hat{\bar{H}}$ and survival, and estimated statistical power as described in the main text.

Power analysis for effects of heterozygosity on patch extinction

Our analyses of power to detect effects of heterozygosity on patch extinction were designed to account for both process and error variance in our estimates of average patch heterozygosity ($\hat{\bar{H}}_P$). We assumed that real patch average heterozygosity $\bar{H}$p was equivalent to $\hat{\bar{H}}_P$. We therefore modeled patch extinction as a function of $\bar{H}$p as described in the main text. To account for error variance, we simulate an estimate of heterozygosity for each individual ($\hat{\bar{H}}$) in each nest withing a patch as described above in the section on the power analysis for overwinter nest mortality. We then take the average of these as a simulated estimate of the average patch heterozygosity ($\hat{\bar{H}}_P$). For each of 5,000 replicates, we test for a statistical association between patch extinction and $\hat{\bar{H}}_P$ and measure power as described in the main text. The number of patches, the number of nests within patches, and the number of individuals sampled within nests for the power analysis were identical to those in the empirical analyses of patch extinction.

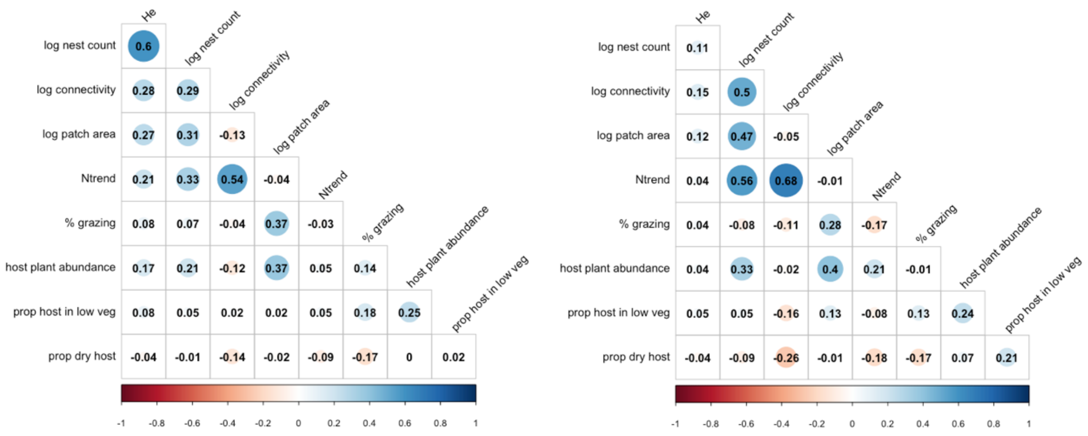

Fig. S1. Pairwise Pearson correlations among covariates for populations (left panel) and nest-level data (right panel).

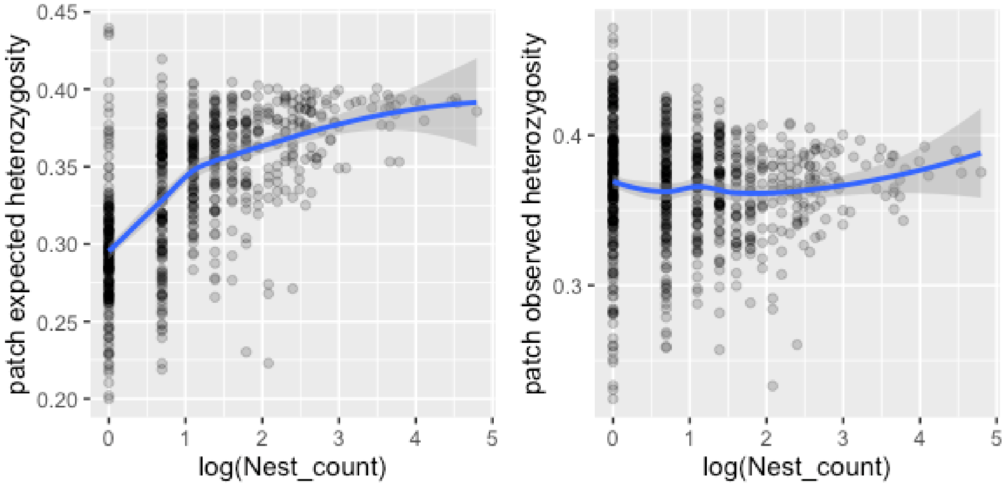

Fig. S2. Relationship between the logarithm of nest count and expected (left panel) and observed (right panel) heterozygosity of populations

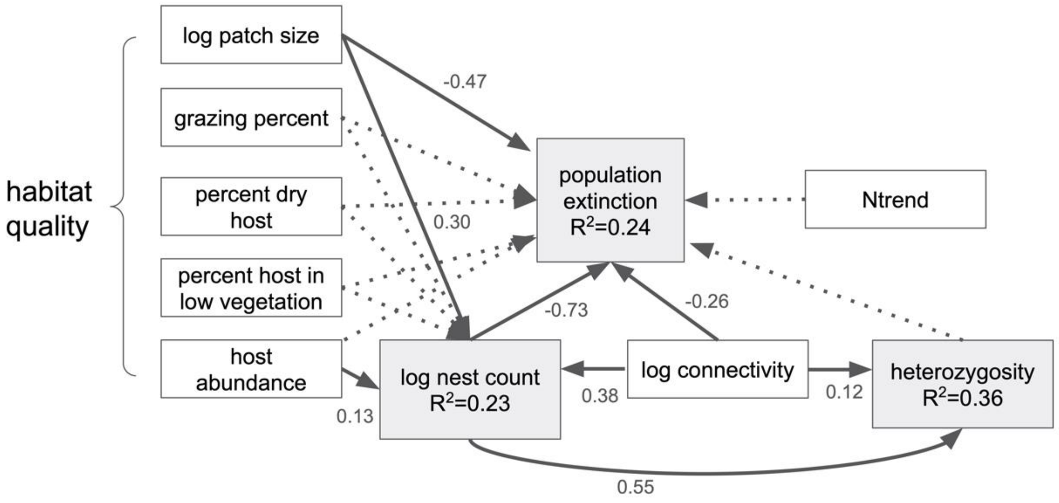

Figure S3. Results from a piecewise structural equation model quantifying direct and indirect effects of heterozygosity, environmental and demographic variables on annual population extinction risk. Solid black lines show significant paths with standardized coefficients along edges. Dotted lines show non-significant paths. Endogenous (dependent) variables are shaded grey and marginal R^2 values are shown. All model coefficients are reported in Table S2

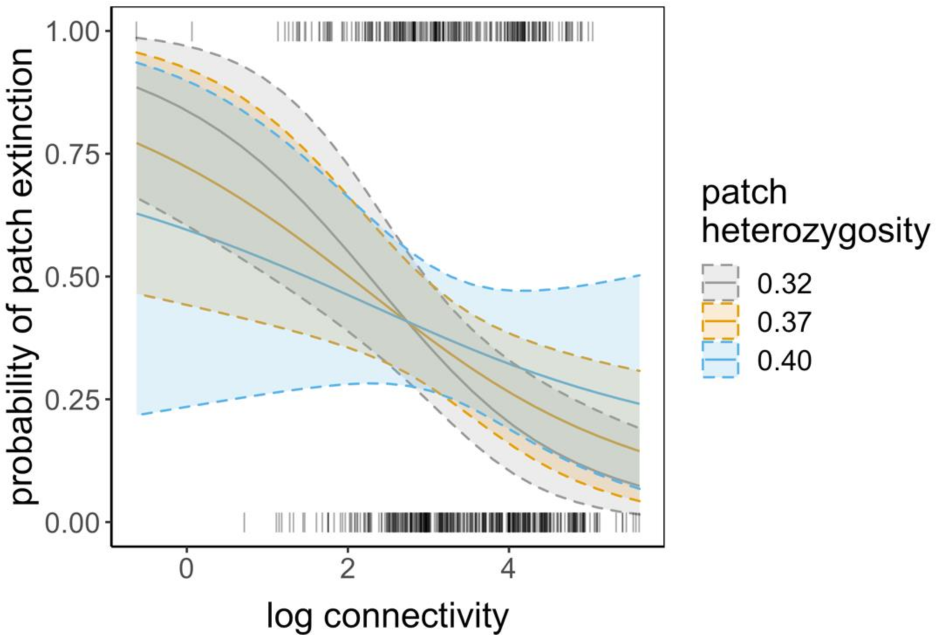

Figure S4. Probability of population extinction as a function of patch connectivity for populations that had low or high observed heterozygosity. Solid lines are posterior mean estimates and dashed lines are 95% credible intervals from model 3 in Table S3

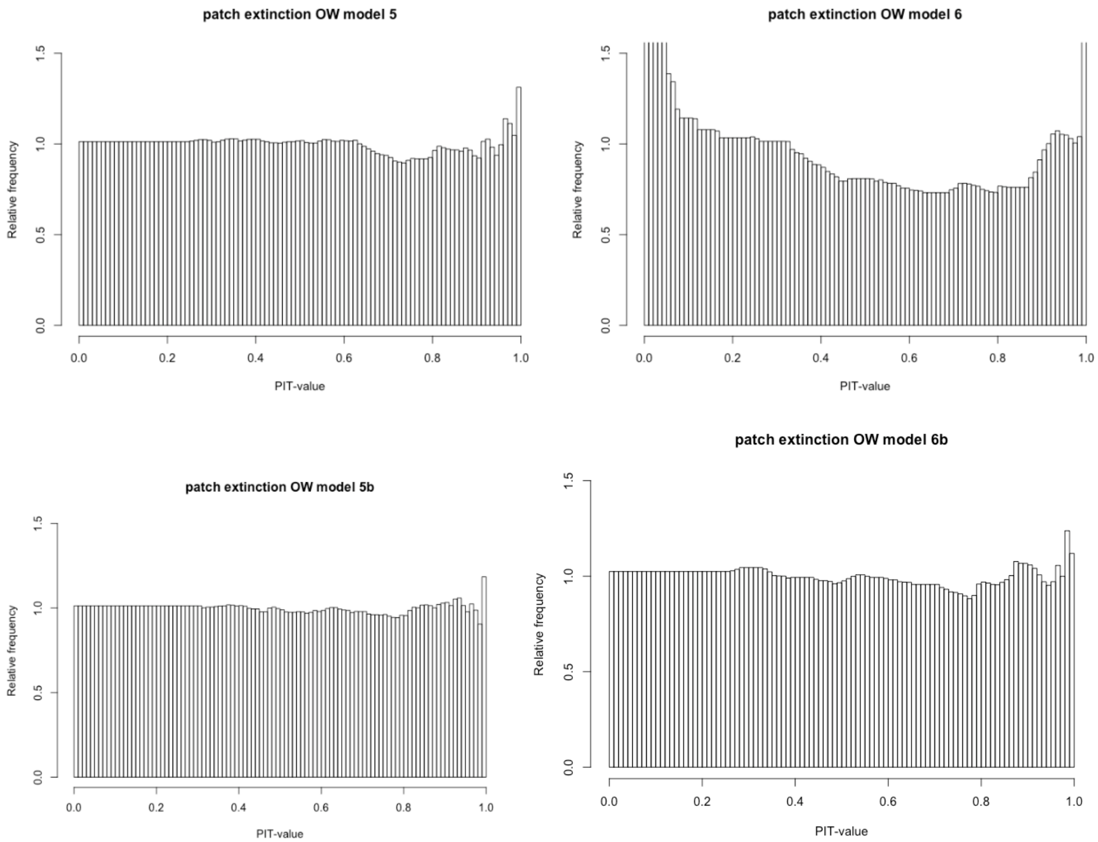

Figure S5. Histograms of PIT values for models 5 and 6 for overwintering population extinction. The top panel shows full models with signs of overfitting. The bottom panel shows models with covariates with small effect sizes removed. Values were adjusted for discrete data using the transformation described in Czado et al 2009. Uniform histograms indicate a good model fit.

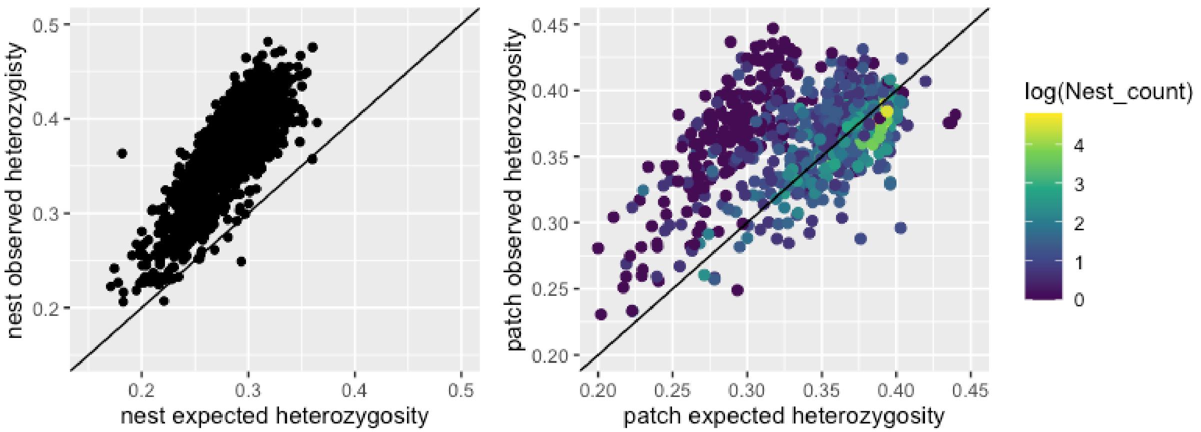

Figure S6. Scatterplots showing correlations between expected and observed heterozygosity for nests (left panel) and populations (right panel). Lines show 1:1 relationship between the two measures. Point colour for population heterozygosity shows the logarithm of nest count. The Pearson correlation is r=0.82 for nests and r=0.44 for populations.

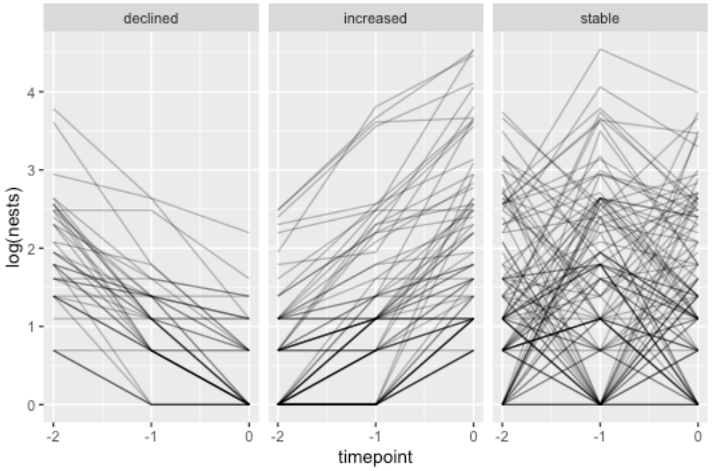

Figure S7. Population trends from year t-2 to t for patches included in model 6. Timepoint 0 is time t. Each line represents the trend for a single data point in the model, with transparency indicating how many times a particular patch was included in the model (e.g. nests from the same patch).

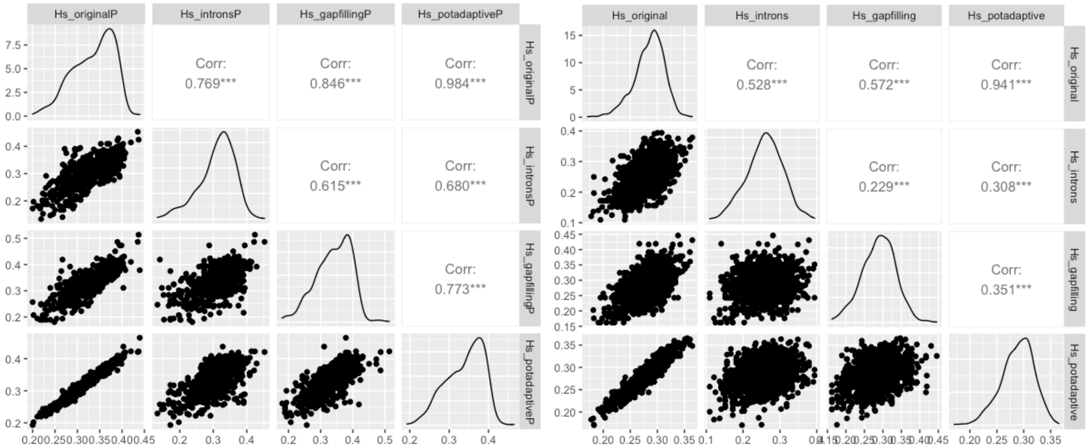

Figure S8. Pairwise Pearson correlation coefficients among heterozygosity values calculated using different subsets of the available SNPs. The left panel shows correlations for patch-level heterozygosity and the right panel show correlations for nest-level heterozygosity.

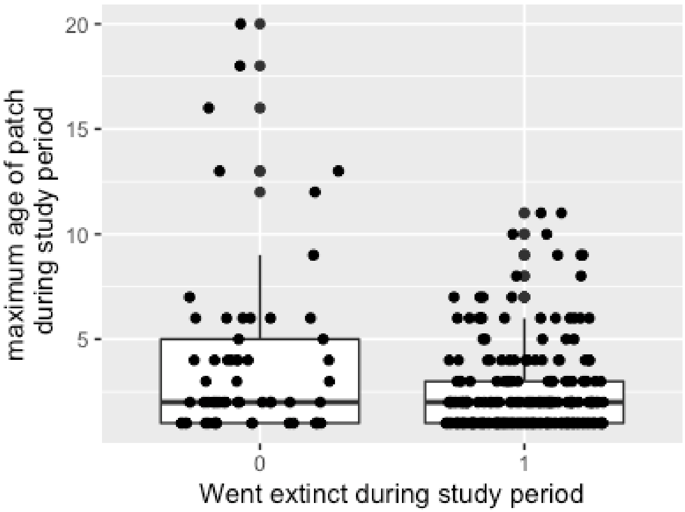

Figure S9. Maximum patch age (i.e. number of years a patch has been continuously occupied) during the study period was higher amongst those patches that persisted during our study than those that went extinct (Poisson generalised linear model, intercept=1.3, beta[extinction]=-0.5, se=0.07, t-value=-6.7, p<0.001) . However, given that relatively few patches persisted more than a few years and many of those that persisted longer than went extinct during our study period all patches were retained in analysis regardless of age of occupancy.

Table S1. Standardized posterior mean estimates, standard deviations (sd) and 95% credibility intervals (CI) for the parameters of the models fitted to annual population extinction

	model 1			model 2			model 3			model 4			model 5			model 6		
Covariate	mean	sd	95% CI	mean	sd	95% CI	mean	sd	95% CI	mean	sd	95% CI	mean	sd	95% CI	mean	sd	95% CI
Intercept	-0.21	0.30	[-0.76,0.44]	**-0.73**	**0.24**	**[-1.2,-0.26]**	**-0.61**	**0.25**	**[-1.1,-0.12]**	**-0.42**	**0.19**	**[-0.79,-0.04]**	**-1.22**	**0.30**	**[-1.8,-0.64]**	**-1.35**	**0.40**	**[-2.2,-0.57]**
expected heterozygosity	**-0.55**	**0.10**	**[-0.75,-0.35]**	0.002	0.13	[-0.25,0.26]	-0.16	0.16	[-0.47,0.15]									
log nest count				**-0.71**	**0.15**	**[-1.0,-0.41]**	**-0.65**	**0.16**	**[-0.97,-0.33]**	**-0.70**	**0.15**	**[-1.0,-0.42]**	**-0.80**	**0.23**	**[-1.3,-0.36]**	**-0.74**	**0.31**	**[-1.4,-0.15]**
log patch area				**-0.53**	**0.14**	**[-0.82,-0.27]**	**-0.52**	**0.14**	**[-0.80,-0.26]**	**-0.51**	**0.13**	**[-0.78,-0.26]**	**-0.50**	**0.19**	**[-0.88,-0.15]**	**-0.51**	**0.25**	**[-1.0,-0.05]**
Ntrend				-0.17	0.22	[-0.59,0.27]	-0.20	0.22	[-0.64,0.24]	-0.12	0.12	[-0.36,0.12]	-0.34	0.30	[-0.92,0.25]	-0.32	0.42	[-1.2,0.47]
log connectivity				**-0.55**	**0.19**	**[-0.95,-0.19]**	**-0.46**	**0.20**	**[-0.88,-0.09]**	**-0.33**	**0.13**	**[-0.59,-0.08]**	-0.23	0.27	[-0.78,0.28]	0.04	0.35	[-0.64,0.75]
host abundance				-0.21	0.12	[-0.46,0.02]	-0.21	0.12	[-0.45,0.03]	-0.10	0.11	[-0.32, 0.11]	0.13	0.17	[-0.20,0.46]	0.25	0.24	[-0.21,0.73]
host in low vegetation				-0.05	0.11	[-0.27,0.16]	-0.04	0.11	[-0.26,0.17]	-0.12	0.10	[-0.32,0.08]	-0.18	0.16	[-0.49,0.13]	-0.16	0.22	[-0.60,0.26]
percentage of dry host				-0.07	0.12	[-0.30,0.16]	-0.09	0.12	[-0.33,0.14]	-0.06	0.10	[-0.26,0.13]	-0.12	0.18	[-0.48,0.22]	-0.20	0.25	[-0.71,0.26]
percentage of grazing				0.22	0.12	[-0.004,0.46]	0.23	0.12	[-0.001,0.46]	0.21	0.11	[-0.01,0.43]	0.24	0.17	[-0.09,0.59]	0.31	0.24	[-0.16,0.80]
He:log nest count							**-0.32**	**0.15**	**[-0.63,-0.03]**									
He:log connectivity							0.11	0.11	[-0.11,0.34]									
He:Year2007-08										-0.30	0.25	[-0.80,0.18]						
He:Year2008-09										-0.04	0.31	[-0.66,0.57]						
He:Year2009-10										-0.17	0.24	[-0.66,0.30]						
He:Year2010-11										0.17	0.27	[-0.37,0.70]						
He:Year2011-12										0.24	0.22	[-0.19,0.67]						
He:Year2012-13										0.20	0.22	[-0.23,0.64]						
He:declined													0.11	0.23	[-0.34,0.56]	0.12	0.37	[-0.59,0.85]
He:increased													0.09	0.32	[-0.52,0.73]	-0.16	0.50	[-1.1,0.82]
He:stable													0.23	0.33	[-0.40,0.90]	-0.22	0.32	[-0.84,0.40]

standard deviation of patch random effect	0.66	0.21	[0.32,1.1]	0.58	0.20	[0.24,1.0]	0.55	0.20	[0.22,1.0]	0.55	0.19	[0.23,0.96]	0.41	0.25	[0.07,0.98]	0.44	0.33	[0.05,1.3]
standard deviation of spatial random effect	0.99	0.22	[0.62,1.5]	1.06	0.24	[0.65,1.6]	1.06	0.25	[0.65,1.6]	0.65	0.33	[0.21,1.5]	1.21	0.35	[0.64,2.0]	1.37	0.44	[0.67,2.4]
range of spatial random effect (km)	10.06	5.14	[3.3,23]	12.59	5.82	[4.7,27]	12.67	5.88	[4.7,27]	3.65	3.43	[0.60,12.7]	0.35	7.45	[2.1,30]	10.20	6.63	[2.3,27.3]
rho	0.41	0.25	[-0.13,0.81]	-0.23	0.26	[-0.68,0.33]	-0.24	0.26	[-0.69,0.32]				-0.29	0.33	[-0.82,0.43]	0.01	0.39	[-0.69,0.74]

Table S2. Results from piecewise structural equation modeling. Estimate for individual paths fitted to annual population extinction, expected heterozygosity (He) and log nest count are given. Statistically significant paths are bolded.

Response		Predictor	Estimate	SE	DF	Crit.Value	P-value
population extinction	~	He	0.03	0.12	643	0.2	0.82
		**log nest count**	**-0.73**	**0.14**	**643**	**-5.1**	**<0.001**
		**log connectivity**	**-0.26**	**0.12**	**643**	**-2.1**	**0.03**
		**log patch area**	**-0.47**	**0.13**	**643**	**-3.6**	**<0.001**
		host abundance	-0.06	0.11	643	-0.6	0.56
		host in low vegetation	-0.09	0.10	643	-0.9	0.35
		percentage of dry host plant	-0.02	0.10	643	-0.2	0.85
		percentage of grazing	0.17	0.11	643	1.6	0.12
		Ntrend	-0.13	0.12	643	-1.1	0.27
He	~	**log nest count**	**0.55**	**0.03**	**504**	**264.2**	**<0.001**
		**log connectivity**	**0.12**	**0.03**	**598**	**11.9**	**<0.001**
log nest count	~	**log connectivity**	**0.38**	**0.03**	**634**	**116.8**	**<0.001**
		**log patch area**	**0.30**	**0.05**	**291**	**37.5**	**<0.001**
		**host abundance**	**0.13**	**0.04**	**631**	**12.4**	**<0.001**
		host in low vegetation	0.01	0.04	627	0.2	0.68
		percentage of dry host plant	0.01	0.03	613	0.1	0.75
		percentage of grazing	-0.04	0.04	500	0.8	0.36

Table S3. Standardized posterior mean estimates, standard deviations (sd) and 95% credibility intervals (CI) for the parameters of the models fitted to population extinction with observed heterozygosity instead of expected heterozygosity

	model 1			model 2			model 3			model 4			model 5			model 6		
Covariate	mean	sd	95% CI	mean	sd	95% CI	mean	sd	95% CI	mean	sd	95% CI	mean	sd	95% CI	mean	sd	95% CI
Intercept	-0.02	0.34	[-0.63,0.73]	**-0.73**	**0.23**	**[-1.2,-0.26]**	**-0.75**	**0.24**	**[-1.2,-0.3]**	**-0.42**	**0.18**	**[-0.78,-0.04]**	**-1.11**	**0.30**	**[-1.7,-0.49]**	**-1.26**	**0.44**	**[-2.1,-0.39]**
observed heterozygosity	-0.01	0.10	[-0.22,0.19]	0.06	0.10	[-0.14,0.26]	0.14	0.13	[-0.11,0.41]									
log nest count				**-0.70**	**0.13**	**[-0.96,-0.45]**	**-0.70**	**0.13**	**[-0.97,-0.45]**	**-0.67**	**0.12**	**[-0.91,-0.42]**	**-0.70**	**0.18**	**[-1.1,-0.35]**	**-0.82**	**0.25**	**[-1.3,-0.35]**
log patch area				**-0.54**	**0.14**	**[-0.82,-0.28]**	**-0.55**	**0.14**	**[-0.83,-0.15]**	**-0.51**	**0.13**	**[-0.78,-0.26]**	**-0.51**	**0.18**	**[-0.88,-0.17]**	**-0.49**	**0.23**	**[-0.97,-0.05**
Ntrend				-0.16	0.22	[-0.59,0.27]	-0.18	0.22	[-0.62,0.28]	-0.08	0.12	[-0.32,0.17]	-0.32	0.30	[-0.9,0.28]	-0.31	0.43	[-1.2,0.49]
log connectivity				**-0.56**	**0.19**	**[-0.96,-0.2]**	**-0.52**	**0.20**	**[-0.94,-0.26]**	**-0.36**	**0.13**	**[-0.63,-0.11]**	**-0.32**	**0.27**	**[-0.88,0.18]**	-0.02	0.35	[-0.72,0.69
host abundance				-0.22	0.12	[-0.46,0.02]	-0.23	0.12	[-0.48,0.007]	-0.11	0.11	[-0.33,0.1]	0.10	0.16	[-0.21,0.42]	**0.24**	**0.24**	**[0.21,0.72]**
host in low vegetation				-0.05	0.11	[-0.27,0.16]	-0.04	0.11	[-0.26,0.17]	-0.11	0.10	[-0.31,0.08]	-0.17	0.15	[-0.47,0.13]	-0.14	0.22	[-0.57,0.29]
percentage of dry host				-0.07	0.12	[-0.3,0.16]	-0.10	0.12	[-0.34,0.13]	-0.06	0.10	[-0.25,0.14]	-0.08	0.18	[-0.45,0.25]	-0.21	0.25	[-0.72,0.25]
percentage of grazing				0.23	0.12	[-0.001,0.46]	0.22	0.12	[-0.01,0.46]	0.22	0.11	[-0.002,0.44]	0.18	0.17	[-0.15,0.52]	0.21	0.24	[-0.25,0.68]
Ho:log nest count							0.08	0.14	[-0.20,0.35]									
Ho:log connectivity							**0.20**	**0.10**	**[0.004,0.41]**									
Ho:Year2007-08										-0.12	0.23	[-0.59,0.33]						
Ho:Year2008-09										-0.01	0.36	[-0.72,0.71]						
Ho:Year2009-10										-0.08	0.20	[-0.48,0.30]						
Ho:Year2010-11										0.28	0.27	[-0.23,0.81]						
Ho:Year2011-12										0.06	0.21	[-0.34,0.48]						
Ho:Year2012-13										0.23	0.19	[-0.13,0.61]						
Ho:declined													**0.79**	**0.25**	**[0.33,1.3]**	0.38	0.32	[-0.22,1.04]
Ho:increased													**0.56**	**0.27**	**[0.04,1.1]**	0.06	0.40	[-0.72,0.84]
Ho:stable													0.38	0.26	[-0.09,0.91]	**0.68**	**0.33**	**[0.07,1.4]**
standard deviation of patch random effect	0.83	0.21	[0.47,1.3]	0.59	0.20	[0.25,1.0]	0.56	0.20	[0.23,1.0]	0.55	0.19	[0.23,0.96]	0.34	0.21	[0.05,0.84]	0.42	0.32	[0.05,1.2]
standard deviation of spatial random effect	1.19	0.26	[0.75,1.8]	1.05	0.24	[0.65,1.6]	1.08	0.25	[0.66,1.6]	0.66	0.35	[0.2,1.5]	1.26	0.39	[0.64,2.1]	1.46	0.47	[0.72,2.6]
range of spatial random effect (km)	6.69	3.71	[2.1,16.2]	12.57	5.77	[4.7,27]	11.93	5.57	[4.5,26]	3.41	3.21	[0.58,11.9]	11.60	8.04	[2.6,33]	10.65	6.84	[2.6,28]
rho	0.57	0.19	[0.13,0.86]	-0.24	0.26	[-0.69,0.32]	-0.22	0.26	[-0.67,0.33]				-0.25	0.34	[-0.81,0.47]	0.12	0.37	[-0.58,0.78]

Table S4. Standardized posterior mean estimates, standard deviations (sd) and 95% credibility intervals (CI) for the parameters of the models fitted to overwintering population extinction

	model 1			model 2			model 3			model 4			model 5			model 6		
Covariate	mean	sd	95% CI	mean	sd	95% CI	mean	sd	95% CI	mean	sd	95% CI	mean	sd	95% CI	mean	sd	95% CI
Intercept	**-1.95**	**0.25**	**[-2.5,-1.5]**	**-2.38**	**0.28**	**[-3.0,-1.9]**	**-2.33**	**0.29**	**[-3.0,-1.8]**	**-2.38**	**0.29**	**[-3.0,-1.9]**	**-3.38**	**0.66**	**[-4.9,-2.3]**	**-5.50**	**2.31**	**[-11.6,-3.0]**
expected heterozygosity	**-0.82**	**0.14**	**[-1.1,-0.55]**	-0.19	0.18	[-0.56,0.16]	-0.49	0.26	[-0.99,0.03]									
log nest count				**-1.56**	**0.30**	**[-2.2,-1.0]**	**-1.63**	**0.31**	**[-2.3,-1.1]**	**-1.53**	**0.28**	**[-2.1,-1.0]**	**-1.80**	**0.49**	**[-2.9,-0.92]**	**-2.50**	**1.44**	**[-6.3,-0.51]**
log patch area				0.13	0.18	[-0.23,0.49]	0.14	0.18	[-0.21,0.51]	0.18	0.19	[-0.19,0.55]						
Ntrend				-0.09	0.29	[-0.67,0.50]	-0.13	0.29	[-0.69,0.44]	-0.16	0.21	[-0.57,0.26]						
log connectivity				0.20	0.22	[-0.24,0.65]	0.32	0.25	[-0.16,0.82]	0.40	0.22	[-0.02,0.85]	0.22	0.32	[-0.40,0.85]	0.67	0.64	[-0.47,2.2]
host abundance				-0.21	0.16	[-0.54,0.10]	-0.21	0.16	[-0.53,0.11]	-0.20	0.16	[-0.52,0.11]						
host in low vegetation				0.02	0.15	[-0.27,0.32]	0.02	0.15	[-0.28,0.32]	0.04	0.15	[-0.26,0.34]						
percentage of dry host				-0.02	0.16	[-0.36,0.29]	-0.04	0.16	[-0.38,0.27]	0.02	0.16	[-0.30,0.33]						
percentage of grazing				0.02	0.17	[-0.32,0.34]	0.04	0.17	[-0.30,0.37]	0.00	0.17	[-0.33,0.33]						
He:log nest count							-0.44	0.29	[-1.0,0.11]									
He:log connectivity							0.12	0.17	[-0.21,0.46]									
He:Year2009-10										-0.06	0.29	[-0.61,0.51]						
He:Year2010-11										**-0.77**	**0.35**	**[-1.5,-0.10]**						
He:Year2011-12										0.25	0.28	[-0.29,0.81]						
He:Year2012-13										-0.54	0.28	[-1.1,0.002]						
He:declined													-0.63	0.37	[-1.4,0.05]	**-1.30**	**0.78**	**[-3.2,-0.06]**
He:increased													-0.31	0.58	[-1.4,0.88]	-1.09	1.25	[-4.0,1.2]
He:stable													-0.31	0.42	[-1.1,0.51]	-0.55	0.69	[-2.0,0.76]
standard deviation of patch random effect	0.51	0.28	[0.12,1.2]	0.49	0.30	[0.10,1.2]	0.46	0.28	[0.09,1.1]	0.37	0.25	[0.05,0.96]	0.51	0.42	[0.06,1.6]	1.26	1.42	[0.12,5.1]
standard deviation of spatial random effect	0.69	0.33	[0.21,1.5]	0.85	0.34	[0.34,1.7]	0.80	0.35	[0.29,1.7]	0.99	0.53	[0.30,2.3]	1.27	0.68	[0.35,2.9]	1.44	1.00	[0.22,3.9]

range of spatial random effect (km)	7.65	10.19	[0.60,33]	9.22	12.95	[0.71,42]	9.27	14.64	[0.53,45]	2.20	1.53	[0.55,6.2]	4.30	4.37	[0.74,15.7]	4.72	4.24	[0.78,15.8]
rho	-0.06	0.62	[-0.95,0.96]	-0.55	0.35	[-0.96,0.33]	-0.51	0.40	[-0.96,0.49]				0.35	0.65	[-0.96,0.99]	0.42	0.54	[-0.83,1.0]

Table S5. Standardized posterior mean estimates, standard deviations (sd) and 95% credibility intervals (CI) for the parameters of the models fitted to overwintering population extinction for models 5 and 6 with all covariates included

	model 5			model 6		
Covariate	mean	sd	95% CI	mean	sd	95% CI
Intercept	**-4.24**	**1.48**	**[-9.9,-2.7]**	**-17.9**	**6.94**	**[-37,-6.5]**
log nest count	**-2.08**	**0.80**	**[-4.2,-0.94]**	**-8.67**	**3.86**	**[-18,-2.6]**
log patch area	0.13	0.38	[-0.63,0.86]	2.87	1.80	[-0.01,6.9]
Ntrend	-1.18	0.94	[-3.6,0.08]	-1.80	2.08	[-6.8,1.4]
log connectivity	0.49	0.51	[-0.35,1.6]	2.56	1.83	[-0.47,6.7]
host abundance	-0.04	0.32	[-0.68,0.56]	-0.69	1.27	[-3.3,1.7]
host in low vegetation	0.39	0.33	[-0.21,1.1]	2.26	1.56	[-0.17,5.9]
percentage of dry host	-0.67	0.47	[-1.9,0.02]	-0.98	1.51	[-4.6,1.4]
percentage of grazing	-0.51	0.40	[-1.4,0.16]	-1.8	1.5	[-5.0,0.66]
He:declined	**-0.96**	**0.53**	**[-2.2,-0.12]**	**-3.7**	**2.0**	**[-8.4,-0.58]**
He:increased	-0.07	0.72	[-1.4,1.4]	-4.9	3.2	[-12,0.29]
He:stable	-0.29	0.55	[-1.4,0.72]	-0.47	1.4	[-3.3,2.4]
standard deviation of patch random effect	1.9	1.9	[0.27,7.2]	3.4	2.2	[0.68,9.1]
standard deviation of spatial random effect	2.6	1.4	[0.75,5.9]	3.9	2.2	[1.0,9.5]
range of spatial random effect (km)	2.7	1.5	[0.88,6.5]	4.5	2.5	[1.3.10.9]
rho	-0.32	0.42	[-0.91,0.60]	0.29	0.51	[-0.79,0.95]

Table S6. Standardized posterior mean estimates, standard deviations (sd) and 95% credibility intervals (CI) for the parameters of the models fitted to overwintering population extinction with observed heterozygosity instead of expected heterozygosity

	model 1			model 2			model 3			model 4			model 5			model 6		
Covariate	mean	sd	95% CI	mean	sd	95% CI	mean	sd	95% CI	mean	sd	95% CI	mean	sd	95% CI	mean	sd	95% CI
Intercept	**-1.77**	**0.23**	**[-2.3,-1.3]**	**-2.39**	**0.27**	**[-3.0,-1.9]**	**-2.41**	**0.29**	**[-3.0,-1.9]**	**-2.35**	**0.28**	**[-3.0,-1.8]**	**-3.21**	**0.55**	**[-4.5,-2.3]**	**-5.21**	**1.90**	**[-10.2,-2.9]**
observed heterozygosity	0.03	0.13	[-0.22,0.29]	0.03	0.13	[-0.22,0.29]	0.06	0.26	[-0.43,0.59]									
log nest count				**-1.72**	**0.26**	**[-2.2,-1.2]**	**-1.76**	**0.27**	**[-2.3,-1.3]**	**-1.61**	**0.25**	**[-2.1,-1.1]**	**-2.35**	**0.47**	**[-3.4,-1.5]**	**-3.13**	**1.25**	**[-6.3,-1.4]**
log patch area				0.11	0.18	[-0.25,0.47]	0.11	0.18	[-0.25,0.48]	0.11	0.19	[-0.25,0.47]						
Ntrend				-0.06	0.29	[-0.63,0.52]	-0.04	0.30	[-0.63,0.57]	-0.19	0.21	[-0.60,0.21]						
log connectivity				0.14	0.22	[-0.30,0.57]	0.12	0.23	[-0.33,0.57]	0.33	0.21	[-0.07,0.76]	0.20	0.27	[-0.32,0.75]	0.42	0.56	[-0.64,1.6]
host abundance				-0.22	0.16	[-0.54,0.09]	-0.22	0.16	[-0.55,0.09]	-0.21	0.16	[-0.52,0.10]						
host in low vegetation				0.01	0.15	[-0.29,0.31]	0.01	0.15	[-0.29,0.31]	0.01	0.15	[-0.28,0.31]						
percentage of dry host				-0.02	0.17	[-0.36,0.29]	-0.02	0.17	[-0.35,0.30]	-0.02	0.16	[-0.35,0.29]						
percentage of grazing				0.02	0.17	[-0.31,0.35]	0.02	0.17	[-0.31,0.35]	0.00	0.17	[-0.34,0.34]						
Ho:log nest count							0.07	0.26	[-0.43,0.6]									
Ho:log connectivity							-0.13	0.14	[-0.41,0.15]									
Ho:Year2009-10										0.04	0.23	[-0.40, 0.49]						
Ho:Year2010-11										0.61	0.44	[-0.17,1.5]						
Ho:Year2011-12										-0.32	0.25	[-0.82,0.17]						
Ho:Year2012-13										0.00	0.22	[-0.43,0.44]						
Ho:declined													-0.07	0.28	[-0.62,0.47]	-0.56	0.45	[-1.5,0.24]
Ho:increased													0.72	0.57	[-0.34,1.9]	0.59	0.86	[-1.1,2.3]
Ho:stable													-0.13	0.29	[-0.69,0.44]	-0.32	0.51	[-1.4,0.65]
standard deviation of patch random effect	0.64	0.28	[0.20,1.3]	0.43	0.26	[0.08,1.0]	0.48	0.29	[0.10,1.2]	0.39	0.25	[0.06,1.0]	0.60	0.44	[0.1,1.7]	0.93	0.97	[0.09,3.6]
standard deviation of spatial random effect	1.05	0.60	[0.28,2.6]	0.85	0.31	[0.37,1.6]	0.87	0.32	[0.38,1.6]	0.94	0.55	[0.25,2.3]	1.22	0.69	[0.30,2.9]	1.56	1.20	[0.22,4.7]
range of spatial random effect (km)	2.28	1.62	[0.54,6.6]	9.65	12.8	[0.71,42]	10.2	13.5	[0.79,44.4]	2.14	1.53	[0.52,6.1]	3.76	3.43	[0.71,12.8]	4.21	3.42	[0.78,13.2]

rho	0.54	0.49	[-0.75,0.99]	-0.58	0.34	[-0.96,0.33]	-0.56	0.33	[-0.95,0.30]				-0.50	0.43	[-0.97,0.57]	0.35	0.56	[-0.86,0.98]

Table S7. Standardized posterior mean estimates, standard deviations (sd) and 95% credibility intervals (CI) for the parameters of the models fitted to overwinter nest mortality

	model 1			model 2			model 3			model 4			model 5			model 6		
Covariate	mean	sd	95% CI	mean	sd	95% CI	mean	sd	95% CI	mean	sd	95% CI	mean	sd	95% CI	mean	sd	95% CI
Intercept	**-0.75**	**0.13**	**[-1.0,-0.5]**	**-0.72**	**0.16**	**[-1.0,-0.41]**	**-0.70**	**0.16**	**[-1.02,-0.39]**	-0.37	0.19	[-0.72,0.05]	**-0.76**	**0.20**	**[-1.1,-0.36]**	**-0.82**	**0.22**	**[-1.26,-0.38]**
expected heterozygosity	-0.02	0.06	[-0.13,0.09]	-0.02	0.06	[-0.13,0.09]	-0.03	0.06	[-0.15,0.08]									
log nest count				**-0.37**	**0.12**	**[-0.59,-0.14]**	**-0.37**	**0.12**	**[-0.59,-0.14]**	-0.11	0.11	[-0.32,0.11]	**-0.46**	**0.14**	**[-0.73,-0.19]**	**-0.37**	**0.16**	**[-0.69,-0.05]**
log patch area				0.17	0.10	[-0.03,0.37]	0.17	0.10	[-0.03,0.37]	0.08	0.11	[-0.13,0.29]	0.15	0.12	[-0.09,0.40]	0.18	0.13	[-0.08,0.45]
Ntrend				**0.41**	**0.19**	**[0.05,0.79]**	**0.44**	**0.19**	**[0.07,0.83]**	**-0.40**	**0.09**	**[-0.59,-0.22]**	0.26	0.23	[-0.19,0.72]	0.44	0.26	[-0.05,0.97]
log connectivity				0.08	0.14	[-0.19,0.35]	0.04	0.14	[-0.24,0.07]	**0.69**	**0.11**	**[0.48,0.91]**	**0.34**	**0.17**	**[0.003,0.67]**	0.03	0.20	[-0.36,0.43]
host abundance				-0.08	0.08	[-0.24,0.07]	-0.09	0.08	[-0.24,0.07]	-0.01	0.08	[-0.15,0.15]	0.06	0.09	[-0.12,0.23]	0.03	0.11	[-0.19,0.24]
host in low vegetation				-0.07	0.07	[-0.22, 0.07]	-0.08	0.07	[-0.22,0.06]	-0.10	0.07	[-0.24,0.04]	0.00	0.09	[-0.18,0.18]	-0.08	0.11	[-0.30,0.14]
percentage of dry host				-0.01	0.08	[-0.17,0.15]	0.00	0.08	[-0.16,0.15]	0.05	0.07	[-0.09,0.18]	-0.03	0.10	[-0.23,0.16]	-0.08	0.12	[-0.31,0.14]
percentage of grazing				0.16	0.09	[-0.006,0.33]	0.16	0.09	[-0.005,0.33]	0.09	0.09	[-0.08,0.27]	0.06	0.11	[-0.16,0.27]	0.22	0.13	[-0.02,0.48]
He:log nest count							-0.02	0.07	[-0.15,0.11]									
He:log connectivity							-0.09	0.07	[-0.21,0.04]									
He:Year2009-10										0.27	0.15	[-0.01,0.56]						
He:Year2010-11										-0.09	0.18	[-0.46,0.27]						
He:Year2011-12										-0.19	0.10	[-0.39,0.006]						
He:Year2012-13										-0.04	0.08	[-0.2,0.12]						
He:declined													**-0.44**	**0.16**	**[-0.76,-0.13]**	**-0.82**	**0.37**	**[-1.6,-0.13]**
He:increased													-0.02	0.08	[-0.17,0.13]	-0.07	0.11	[-0.28,0.13]
He:stable													0.26	0.30	[-0.31,0.87]	-0.12	0.12	[-0.36,0.12]
standard deviation of patch random effect	0.61	0.11	[0.42,0.85]	0.50	0.12	[0.30,0.76]	0.52	0.12	[0.32,0.78]	0.55	0.12	[0.34,0.81]	0.48	0.14	[0.26,0.80]	0.35	0.13	[0.14,0.65]
standard deviation of spatial random effect	0.71	0.17	[0.45,1.1]	0.77	0.17	[0.50,1.1]	0.78	0.16	[0.5,1,14]	0.89	0.38	[0.37,1.8]	0.75	0.21	[0.42,1.2]	0.77	0.23	[0.42,1.3]
range of spatial random effect (km)	14.07	7.28	[5.0,32.7]	11.98	5.90	[4.4,26.9]	11.56	5.68	[4.3,25.9]	2.16	1.41	[0.57,5.8]	13.23	8.53	[3.4,35.4]	22.94	17.59	[5.1,69.1]

rho	-0.71	0.18	[-0.95,-0.24]	-0.72	0.17	[-0.94,-0.29]	-0.72	0.17	[-0.94,-0.29]				-0.63	0.24	[-0.94,-0.03]	-0.61	0.24	[-0.9,-0.01]

Table S8. Standardized posterior mean estimates, standard deviations (sd) and 95% credibility intervals (CI) for the parameters of the models fitted to population extinction for site A

	model 1			model 2			model 3			model 4			model 5			model 6		
Covariate	mean	sd	95% CI	mean	sd	95% CI	mean	sd	95% CI	mean	sd	95% CI	mean	sd	95% CI	mean	sd	95% CI
Intercept	-0.14	0.44	[-0.92,0.86]	**-0.83**	**0.35**	**[-1.5,-0.05]**	-0.66	0.35	[-1.3,0.08]	-0.50	0.32	[-1.1,0.22]	**-1.22**	**0.30**	**[-1.8,-0.64]**	**-1.35**	**0.40**	**[-0.22,-0.57]**
expected heterozygosity	**-0.41**	**0.11**	**[-0.64,-0.19]**	0.20	0.15	[-0.09,0.50]	-0.01	0.19	[-0.37,0.36]									
log nest count				**-0.86**	**0.19**	**[-1.2,-0.5]**	**-0.79**	**0.20**	**[-1.2,-0.41]**	**-0.83**	**0.18**	**[-1.2,-0.48]**	**-0.80**	**0.23**	**[-1.3,-0.36]**	**-0.74**	**0.31**	**[-1.4,-0.15]**
log patch area				**-0.47**	**0.16**	**[-0.8,-0.17]**	**-0.45**	**0.16**	**[-0.78,-0.14]**	**-0.47**	**0.16**	**[-0.79,-0.17]**	**-0.50**	**0.19**	**[-0.88,-0.15]**	**-0.51**	**0.25**	**[-1.0,-0.05]**
Ntrend				-0.02	0.28	[-0.57,0.51]	-0.10	0.29	[-0.67,0.46]	-0.07	0.15	[-0.37,0.22]	-0.34	0.30	[-0.92,0.25]	-0.32	0.42	[-1.2,0.46]
log connectivity				**-0.68**	**0.25**	**[-1.2,-0.22]**	**-0.55**	**0.26**	**[-1.1,-0.07]**	**-0.36**	**0.15**	**[-0.67,-0.07]**	-0.23	0.27	[-0.78,0.28]	0.04	0.35	[-0.64,0.75]
host abundance				-0.19	0.14	[-0.47,0.09]	-0.18	0.14	[-0.46,0.1]	-0.06	0.13	[-0.31,0.19]	0.13	0.17	[-0.20,0.46]	0.25	0.24	[-0.21,0.73]
host in low vegetation				-0.14	0.13	[-0.39,0.11]	-0.13	0.13	[-0.38,0.12]	-0.20	0.12	[-0.44,0.02]	-0.18	0.16	[-0.49,0.13]	-0.16	0.22	[-0.60,0.26]
percentage of dry host				-0.19	0.14	[-0.46,0.07]	-0.22	0.14	[-0.5,0.04]	-0.21	0.12	[-0.45,0.03]	-0.12	0.18	[-0.48,0.22]	-0.20	0.25	[-0.71,0.26]
percentage of grazing				0.06	0.14	[-0.21,0.32]	0.05	0.14	[-0.22,0.32]	0.09	0.13	[-0.17,0.35]	0.24	0.17	[-0.09,0.59]	0.31	0.24	[-0.16,0.80]
He:log nest count							**-0.40**	**0.18**	**[-0.77,-0.05]**									
He:log connectivity							0.09	0.13	[-0.16,0.34]									
He:Year2007-08										-0.23	0.30	[-0.83,0.35]						
He:Year2008-09										0.55	0.54	[-0.49,1.6]						
He:Year2009-10										0.08	0.26	[-0.43,0.58]						
He:Year2010-11										0.34	0.29	[-0.21,0.92]						
He:Year2011-12										0.27	0.24	[-0.21,0.75]						
He:Year2012-13										0.37	0.26	[-0.13,0.88]						
He:declined													0.11	0.23	[-0.34,0.56]	0.12	0.37	[-0.59,0.85]
He:increased													0.09	0.32	[-0.52,0.73]	-0.16	0.50	[-1.1,0.82]
He:stable													0.23	0.33	[-0.40,0.90]	-0.22	0.32	[-0.83,0.40]
standard deviation of patch random effect	0.69	0.23	[0.33,1.2]	0.63	0.24	[0.26,1.2]	0.55	0.23	[0.19,1.1]	0.57	0.22	[0.21,1.0]	0.41	0.25	[0.07,0.98]	0.44	0.33	[0.05,1.2]

standard deviation of spatial random effect	1.05	0.27	[0.6,1.7]	1.19	0.33	[0.66,2.0]	1.19	0.33	[0.66,1.9]	0.58	0.23	[0.23,1.1]	1.21	0.35	[0.64,2.0]	1.37	0.44	[0.67,2.4]
range of spatial random effect (km)	10.46	7.66	[2.5,30.7]	16.45	10.77	[4.7,44.8]	16.06	10.64	[4.5,44.0]	8.01	9.20	[0.97,32]	10.52	7.45	[2.1,29.9]	10.23	6.63	[2.3,27.3]
rho	0.60	0.22	[0.08,0.9]	-0.09	0.33	[-0.66,0.58]	-0.11	0.33	[-0.67,0.56]				-0.29	0.33	[-0.82,0.43]	0.01	0.39	[-0.68,0.74]

Table S9. Standardized posterior mean estimates, standard deviations (sd) and 95% credibility intervals (CI) for the parameters of the models fitted to overwinter population extinction for site A

	model 1			model 2			model 3			model 4			model 5			model 6		
Covariate	mean	sd	95% CI	mean	sd	95% CI	mean	sd	95% CI	mean	sd	95% CI	mean	sd	95% CI	mean	sd	95% CI
Intercept	**-1.95**	**0.29**	**[-2.6,-1.4]**	**-2.46**	**0.34**	**[-3.2,-1.9]**	**-2.39**	**0.35**	**[-3.1,-1.8]**	**-2.52**	**0.37**	**[-3.3,-1.9]**	**-3.38**	**0.66**	**[-4.9,-2.3]**	**-5.50**	**2.31**	**[-11.6,-3.0]**
expected heterozygosity	**-0.85**	**0.15**	**[-1.1,-0.55]**	-0.20	0.19	[-0.59,0.18]	-0.52	0.29	[-1.1,0.06]									
log nest count				**-1.59**	**0.32**	**[-2.3,-1.0]**	**-1.65**	**0.34**	**[-2.3,-1.0]**	**-1.65**	**0.33**	**[-2.3,-1.0]**	**-1.80**	**0.49**	**[-2.9,-0.92]**	**-2.50**	**1.44**	**[-6.3,-0.51]**
log patch area				0.17	0.21	[-0.23,0.58]	0.18	0.21	[-0.22,0.59]	0.21	0.21	[-0.20,0.62]						
Ntrend				-0.24	0.27	[-0.77,0.31]	-0.26	0.28	[-0.80,0.29]	-0.21	0.24	[-0.68,0.26]						
log connectivity				0.17	0.24	[-0.29,0.67]	0.24	0.26	[-0.25,0.76]	0.26	0.24	[-0.2,0.75]	0.22	0.32	[-0.40,0.85]	0.67	0.64	[-0.47,2.2]
host abundance				-0.27	0.18	[-0.64,0.08]	-0.27	0.18	[-0.63,0.09]	-0.28	0.18	[-0.65,0.07]						
host in low vegetation				0.10	0.17	[-0.22,0.44]	0.10	0.17	[-0.22,0.44]	0.11	0.17	[-0.21,0.45]						
percentage of dry host				0.14	0.18	[-0.24,0.48]	0.10	0.18	[-0.28,0.44]	0.18	0.18	[-0.19,0.52]						
percentage of grazing				0.01	0.19	[-0.36,0.37]	0.03	0.19	[-0.34,0.39]	0.01	0.19	[-0.36,0.38]						
He:log nest count							-0.43	0.31	[-1.0,0.17]									
He:log connectivity							0.01	0.18	[-0.33,0.36]									
He:Year2009-10										-0.22	0.31	[-0.83,0.39]						
He:Year2010-11										-0.46	0.34	[-1.1,0.20]						
He:Year2011-12										0.21	0.30	[-0.37,0.80]						
He:Year2012-13										-0.45	0.33	[-1.1,0.19]						
He:declined													-0.63	0.37	[-1.4,0.05]	**-1.30**	**0.78**	**[-3.2,-0.06]**
He:increased													-0.31	0.58	[-1.4,0.88]	-1.09	1.25	[-4.0,1.2]
He:stable													-0.31	0.42	[-1.1,0.51]	-0.55	0.69	[-2.0,0.76]
standard deviation of patch random effect	0.42	0.26	[0.07,1.0]	0.35	0.25	[0.05,0.96]	0.36	0.25	[0.05,0.99]	0.36	0.25	[0.05,0.97]	0.51	0.42	[0.06,1.6]	1.26	1.42	[0.12,5.1]
standard deviation of spatial random effect	0.77	0.35	[0.26,1.6]	1.01	0.51	[0.32,2.2]	0.95	0.49	[0.29,2.2]	1.08	0.54	[0.36,2.4]	1.27	0.68	[0.35,2.9]	1.44	1.00	[0.22,3.9]

range of spatial random effect (km)	5.01 5.35 [0.62,19.0]	2.68 2.05 [0.58,8.1]	2.78 2.21 [0.57,8.6]	2.37 1.67 [0.58,6.7]	4.30 4.37 [0.74,15.7]	4.72 4.24 [0.78,15.8]
rho	0.28 0.53 [-0.81,0.96]	0.43 0.58 [-0.90,0.99]	0.45 0.57 [-0.88,0.99]		0.35 0.65 [-0.96,0.99]	0.42 0.54 [-0.83,0.99]

Table S10. Standardized posterior mean estimates, standard deviations (sd) and 95% credibility intervals (CI) for the parameters of the models fitted to overwinter nest mortality for site A

	model 1			model 2			model 3			model 4			model 5			model 6		
Covariate	mean	sd	95% CI	mean	sd	95% CI	mean	sd	95% CI	mean	sd	95% CI	mean	sd	95% CI	mean	sd	95% CI
Intercept	-0.72	0.17	**[-1.1,-0.38]**	**-0.73**	**0.20**	**[-1.1,-0.33]**	**-0.73**	**0.20**	**[-1.1,-0.33]**	-0.43	0.23	[-0.85,0.08]	-0.76	0.20	**[-1.1,-0.36]**	**-0.82**	**0.22**	**[-1.2,-0.38]**
expected heterozygosity	-0.04	0.06	[-0.16,0.07]	-0.03	0.06	[-0.15,0.08]	-0.04	0.06	[-0.16,0.07]									
log nest count				**-0.34**	**0.12**	**[-0.58,-0.10]**	**-0.34**	**0.12**	**[-0.58,-0.10]**	-0.10	0.12	[-0.32,0.13]	-0.46	0.14	**[-0.73,-0.19]**	**-0.37**	**0.16**	**[-0.69,-0.05]**
log patch area				0.12	0.11	[-0.09,0.33]	0.12	0.11	[-0.09,0.33]	0.05	0.11	[-0.18,0.27]	0.15	0.12	[-0.09,0.40]	0.18	0.13	[-0.08,0.45]
Ntrend				**0.44**	**0.22**	**[0.03,0.88]**	**0.46**	**0.22**	**[0.04,0.90]**	**-0.43**	**0.10**	**[-0.62,-0.24]**	0.26	0.23	[-0.19,0.72]	0.44	0.26	[-0.05,0.97]
log connectivity				0.02	0.16	[-0.30,0.33]	0.00	0.16	[-0.33,0.31]	**0.64**	**0.11**	**[0.42,0.85]**	0.34	0.17	**[0.002,0.67]**	0.03	0.20	[-0.36.0.43]
host abundance				-0.09	0.08	[-0.26,0.07]	-0.09	0.09	[-0.26,0.07]	-0.01	0.08	[-0.17,0.15]	0.06	0.09	[-0.12,0.24]	0.03	0.11	[-0.19,0.24]
host in low vegetation				-0.08	0.08	[-0.23,0.08]	-0.08	0.08	[-0.23,0.07]	-0.07	0.08	[-0.22,0.08]	0.00	0.09	[-0.18,0.18]	-0.08	0.11	[-0.31,0.14]
percentage of dry host				-0.01	0.09	[-0.18,0.16]	0.00	0.09	[-0.18,0.17]	0.06	0.07	[-0.09,0.20]	-0.03	0.10	[-0.23,0.16]	-0.08	0.12	[-0.31,0.14]
percentage of grazing				**0.18**	**0.09**	**[0.001,0.36]**	**0.18**	**0.09**	**[0.002,0.36]**	0.10	0.10	[-0.09,0.29]	0.06	0.11	[-0.16,0.27]	0.22	0.13	[-0.02,0.48]
He:log nest count							-0.01	0.07	[-0.14,0.13]									
He:log connectivity							-0.06	0.06	[-0.19,0.06]									
He:Year2009-10										0.21	0.15	[-0.08,0.51]						
He:Year2010-11										-0.07	0.18	[-0.42,0.28]						
He:Year2011-12										-0.19	0.10	[-0.39,0.01]						
He:Year2012-13										-0.04	0.09	[-0.21,0.13]						
He:declined													**-0.44**	**0.16**	**[-0.76,-0.13]**	**-0.82**	**0.37**	**[-1.6,-0.13]**
He:increased													-0.02	0.08	[-0.17,0.13]	-0.07	0.11	[-0.28,0.13]
He:stable													0.26	0.30	[-0.31,0.87]	-0.12	0.12	[-0.36,0.12]
standard deviation of patch random effect	0.61	0.11	[0.42,0.85]	0.50	0.12	[0.29,0.77]	0.51	0.12	[0.30,0.78]	0.57	0.12	[0.35,0.83]	0.48	0.14	[0.25,0.80]	0.35	0.13	[0.14,0.65]
standard deviation of spatial random effect	0.66	0.15	[0.41,1.0]	0.76	0.17	[0.49,1.1]	0.78	0.17	[0.49,1.1]	0.91	0.37	[0.38,1.8]	0.75	0.21	[0.42,1.2]	0.77	0.23	[0.42,1.3]

range of spatial random effect (km)	11.07 5.67 [4.1,25.7]	10.20 5.11 [3.7,23.3]	10.04 5.00 [3.6,22.9]	2.28 1.54 [0.58,6.3]	13.23 8.53 [3.4,35.4]	22.94 17.59 [5.1,69.1]
rho	-0.48 0.26 [-0.88,0.11]	-0.57 0.22 [-0.89,-0.04]	-0.57 0.22 [-0.89,-0.04]		-0.63 0.24 [-0.94,-0.02]	-0.61 0.24 [-0.93,-0.01]

Table S11. Summary of SNPs used in this study including location information and marker type.

KASP ID	Gene	Chr	Scaffold	Position	Marker type	Annotation
KASP2.101	MCINX3812	1	scaffold1791	57299	Candidate	Alpha-tubulin N-acetyltransferase, Alpha-tubulin N-acetyltransferase
KASP2.102	MCINX6747	1	scaffold2404	32920	Candidate	OCIA domain containing 1, Asrij
KASP2.103	MCINX15187	7	scaffold7849	5756	Candidate	Putative serine-type enodpeptidase, Chymotrypsinogen-like protein 3 (Precursor)
KASP2.108	MCINX9609	22	scaffold337	97709	Candidate	SP14D1, CLIPB5 protein
KASP2.11	MCINX4100	4	scaffold184	37271	Candidate	Muscular protein 20, Muscular protein 20
KASP2.111	MCINX218	2	scaffold1064	194473	Candidate	Cutile protein, putative, CPR65
KASP2.112	MCINX2853	11	scaffold1621	69548	Candidate	Cuticular protein RR-1 motif 34, Cuticular protein RR-1 motif 34
KASP2.13	MCINX4735	20	scaffold1940	38758	Candidate	ATP-dependent RNA helicase DBP2, ARB_07144
KASP2.15	MCINX4756	17	scaffold1945	72755	Candidate	Glyceraldehyde-3-phosphate dehydrogenase 2, Glyceraldehyde-3-phosphate dehydr
KASP2.19	MCINX5165	9	scaffold2016	5755	Candidate	Dehydrogenase/reductase SDR family member 7B, Transmembrane protein 11 (Frag
KASP2.2	MCINX111	17	scaffold1041	122591	Candidate	Polyadenylation factor I subunit 2, CG1109, isoform A
KASP2.21	MCINX5295	19	scaffold2035	16508	Candidate	Tubulin polyglutamylase TTLL13, Ubiquilin-1
KASP2.24	MCINX6287	19	scaffold2265	29552	Candidate	Putative 40S ribosomal protein S6, 40S ribosomal protein S6
KASP2.25	MCINX6493	3	scaffold2325	5112	Candidate	Atg5, Putative uncharacterized protein Porcn
KASP2.26	MCINX6830	16	scaffold2442	149441	Candidate	Alpha-crystallin B chain, Alpha-crystallin B chain
KASP2.28	MCINX7660	18	scaffold266	99797	Candidate	CG10219, AAEL010519
KASP2.30	MCINX7638	26	scaffold2666	34531	Candidate	40S ribosomal protein S16 (Fragment), 40S ribosomal protein S16 (Fragment)
KASP2.31	MCINX7807	18	scaffold2702	43336	Candidate	Putative hypoxia-inducible factor 1 alpha, Hypoxia-inducible factor 1 alpha, hif-1 alph
KASP2.33	MCINX7806	18	scaffold2702	9668	Candidate	Putative hypoxia-inducible factor 1 alpha, Hypoxia inducible factor alpha 1 (Fragmen

**KASP2.5**	MCINX975	6	scaffold1247	17513	Candidate	Alpha-crystallin B chain (Fragment), Heat shock protein hsp20.8
**KASP2.54**	MCINX9373	25	scaffold3283	15219	Candidate	
**KASP2.58**	MCINX9466	13	scaffold3324	67067	Candidate	Signal recognition particle receptor beta subunit, Srprb
**KASP2.60**	MCINX11014	13	scaffold4186	37262	Candidate	Decapping protein 1, Serine/threonine-protein phosphatase dullard homolog
**KASP2.69**	MCINX11838	9	scaffold4652	15924	Candidate	60S ribosomal protein L10 (Fragment), 60S ribosomal protein L10 (Fragment)
**KASP2.73**	MCINX12314	2	scaffold4882	90334	Candidate	Solute carrier family 7 (cationic amino acid transporter, y+ system), member 7, Solu
**KASP2.77**	MCINX13877	NA	scaffold63	27348	Candidate	Serine/threonine-protein kinase PAK 3, P21 protein (Cdc42/Rac)-activated kinase 3
**KASP2.79**	MCINX14130	17	scaffold664	168063	Candidate	Hypothetical protein, RIO kinase 3
**KASP2.8**	MCINX2811	23	scaffold1612	105796	Candidate	Succinate dehydrogenase ubiquinone iron-sulfur subunit, Succinate dehydrogenase
**KASP2.80**	MCINX14129	17	scaffold664	166435	Candidate	RIO kinase 3
**KASP2.83**	MCINX3735	10	scaffold1785	58421	Candidate	Heat shock cognate 70 protein, Heat shock cognate 70 protein
**KASP2.84**	MCINX9942	1	scaffold3568	15483	Candidate	Leucine aminopeptidase-like protein (Fragment), Leucine aminopeptidase-like protei
**KASP2.86**	MCINX9374	25	scaffold3283	24287	Candidate	Glucose-6-phosphate isomerase (Fragment)
**KASP2.94**	MCINX9142	1	scaffold31	114406	Candidate	Death-associated kinase 2
**KASP3.10**	MCINX504	NA	scaffold1138	1991	Candidate	Hypothetical protein
**KASP3.101**	MCINX7843	14	scaffold2707	26367	Candidate	Mismatch repair ATPase Msh2
**KASP3.106**	MCINX8216	9	scaffold2825	36163	Candidate	Tetraspanin D76
**KASP3.107**	MCINX8465	7	scaffold2914	10280	Candidate	Gamma-interferon-inducible lysosomal thiol reductase, putative, SP14D1
**KASP3.108**	MCINX8590	25	scaffold2960	33976	Candidate	40S ribosomal protein S7 (Fragment), 40S ribosomal protein S7
**KASP3.109**	MCINX8601	18	scaffold2966	24907	Candidate	ATP synthase-coupling factor 6, mitochondrial, Mitochondrial ATP synthase coupling
**KASP3.12**	MCINX594	13	scaffold1157	16716	Candidate	NADH dehydrogenase 1 alpha subcomplex subunit 6, BDCG_05250

KASP3.134	MCINX9105	4	scaffold3183	2340	Candidate	
KASP3.137	MCINX9105	4	scaffold3183	3654	Candidate	
KASP3.139	MCINX9288	11	scaffold324	12305	Candidate	Dihydrolipoyl dehydrogenase (Fragment), Lipoamide dehydrogenase-glc
KASP3.144	MCINX9501	22	scaffold333	194407	Candidate	MKIAA0178
KASP3.145	MCINX10010	14	scaffold3593	85685	Candidate	Hexamerin 2, Arylphorin
KASP3.146	MCINX10113	9	scaffold3645	35132	Candidate	Putative C1A cysteine protease (Precursor)
KASP3.148	MCINX12495	7	scaffold4	13851	Candidate	
KASP3.15	MCINX791	22	scaffold1202	145207	Candidate	Hypothetical protein, Hypothetical protein
KASP3.150	MCINX11014	13	scaffold4186	33875	Candidate	Decapping protein 1, Serine/threonine-protein phosphatase dullard homolog
KASP3.154	MCINX11502	9	scaffold451	199852	Candidate	
KASP3.157	MCINX11623	20	scaffold457	64536	Candidate	Aurora kinase C, Putative hydroxypyruvate isomerase
KASP3.162	MCINX11979	NA	scaffold4720	1329	Candidate	Mitosis protein dim1, Mitosis protein dim1
KASP3.166	MCINX13201	11	scaffold563	119263	Candidate	Cuticular protein RR-2 motif 83
KASP3.174	MCINX13974	18	scaffold650	113747	Candidate	Bilin-binding protein
KASP3.176	MCINX14137	11	scaffold6658	26653	Candidate	2-oxoglutarate dehydrogenase, mitochondrial, Nc73EF
KASP3.177	MCINX14336	12	scaffold693	50682	Candidate	Probable 60S ribosomal protein L12, Probable 60S ribosomal protein L12
KASP3.179	MCINX14578	4	scaffold724	43429	Candidate	Putative WD-repeat containing protein
KASP3.18	MCINX1291	1	scaffold1309	42614	Candidate	Tan protein, Dper_GLEANR_20319
KASP3.184	MCINX14721	23	scaffold738	222536	Candidate	Casein kinase I isoform alpha
KASP3.185	MCINX14799	15	scaffold7476	10977	Candidate	Diapausin
KASP3.189	MCINX16170	6	scaffold902	81246	Candidate	

**KASP3.19**	MCINX1502	25	scaffold1358	77019	Candidate	Gpatch1 protein
**KASP3.192**	MCINX16177	15	scaffold903	73713	Candidate	
**KASP3.2**	MCINX66	13	scaffold102	89566	Candidate	Putative enolase protein, Putative enolase protein
**KASP3.21**	MCINX1766	8	scaffold1414	133969	Candidate	Hepatocyte growth factor-regulated tyrosine kinase substrate
**KASP3.22**	MCINX1944	11	scaffold1447	264412	Candidate	Hypothetical protein, Hypothetical protein
**KASP3.27**	MCINX2283	8	scaffold1526	287869	Candidate	Suppressor of cytokine signaling 8
**KASP3.28**	MCINX2345	16	scaffold1538	37774	Candidate	Alpha-methylacyl-CoA racemase, putative, Alpha-methylacyl-CoA racemase, putati[illegible]
**KASP3.34**	MCINX2763	4	scaffold1604	81348	Candidate	Probable molt-inhibiting hormone
**KASP3.39**	MCINX3744	3	scaffold1785	127453	Candidate	Bzd, Bzd-PB
**KASP3.40**	MCINX3744	3	scaffold1785	136985	Candidate	Bzd, Bzd-PB
**KASP3.41**	MCINX3772	6	scaffold1787	205335	Candidate	Gamma-interferon-inducible lysosomal thiol reductase, putative
**KASP3.42**	MCINX3778	6	scaffold1787	303991	Candidate	Hypothetical protein, Hypothetical protein
**KASP3.43**	MCINX3918	17	scaffold1818	48557	Candidate	Seminal fluid protein HACP044
**KASP3.48**	MCINX4069	15	scaffold1841	164407	Candidate	Heat shock protein hsp21.4, CpipJ_CPIJ009817
**KASP3.49**	MCINX4177	9	scaffold1863	121044	Candidate	Beta-catenin, Putative catenin beta 1 (Fragment)
**KASP3.5**	MCINX191	21	scaffold1061	35594	Candidate	Malate dehydrogenase 2, NAD (mitochondrial), Malate dehydrogenase 2, NAD (mit[illegible]
**KASP3.50**	MCINX4198	1	scaffold1865	153252	Candidate	Tyrosine 3-monooxygenase, Tyrosine 3-monooxygenase
**KASP3.51**	MCINX4221	10	scaffold1866	196745	Candidate	Cuticular protein RR-1 motif 5, Cuticular protein RR-1 motif 5
**KASP3.59**	MCINX4792	26	scaffold1951	116146	Candidate	GE12277, CpipJ_CPIJ000246
**KASP3.62**	MCINX5065	8	scaffold2001	103903	Candidate	Phosphatidylinositol transfer protein beta isoform, Phosphatidylinositol transfer protei[illegible]
**KASP3.65**	MCINX5230	20	scaffold2025	177786	Candidate	Conserved domain protein, D-tyrosyl-tRNA(Tyr) deacylase

**KASP3.69**	MCINX5450	10	scaffold2059	45235	Candidate	LYR motif-containing protein 7 (Fragment), LYR motif-containing protein 7 (Fragment
**KASP3.75**	MCINX6401	23	scaffold2301	55107	Candidate	Sarcoplasmic reticulum glycoprotein
**KASP3.77**	MCINX6500	3	scaffold2325	42401	Candidate	60S ribosomal protein L5, 60S ribosomal protein L5
**KASP3.82**	MCINX6833	11	scaffold2445	1620	Candidate	50S ribosomal protein L20
**KASP3.83**	MCINX7059	24	scaffold2519	62701	Candidate	
**KASP3.86**	MCINX7155	7	scaffold2544	2885	Candidate	Putative Ofd1 protein
**KASP3.87**	MCINX7377	4	scaffold2617	40756	Candidate	Glucose dehydrogenase [acceptor], Glucose dehydrogenase [acceptor]
**KASP3.90**	MCINX7425	2	scaffold2631	2943	Candidate	Hypothetical protein
**KASP4.33**	MCINX5469	9	scaffold2061	158845	Candidate	
**KASP4.39**	MCINX6587	14	scaffold2353	55883	Candidate	Putative organic cation transporter
**KASP4.5**	MCINX333	24	scaffold1086	100694	Candidate	
**KASP4.6**	MCINX818	17	scaffold1206	26737	Candidate	Invertebrate LYSozyme, CG6421
**KASP4.9**	MCINX1061	18	scaffold1265	60590	Candidate	Protease inhibitor 6, Protease inhibitor 6
**KASP4.90**	MCINX13417	22	scaffold586	220116	Candidate	Putative fatbody protein 3Rev-G1
**KASP4.92**	MCINX13508	13	scaffold591	135224	Candidate	Mitochondrial cytochrome c oxidase subunit 6A isoform 3, Mitochondrial cytochrome
**KASP5.1**	MCINX149	12	scaffold1051	107967	Candidate	ATP-dependent RNA helicase DBP2, putative, DEAD box ATP-dependent RNA helic
**KASP5.109**	MCINX12085	28	scaffold476	68027	Candidate	Hypothetical protein, Hypothetical protein
**KASP5.112**	MCINX13066	12	scaffold547	75393	Candidate	Hypothetical protein, CpipJ_CPIJ003189
**KASP5.120**	MCINX14916	16	scaffold7580	60317	Candidate	Chemosensory protein, Chemosensory protein 5
**KASP5.126**	MCINX14984	12	scaffold7655	31760	Candidate	MPV17 mitochondrial membrane protein-like 2, Hypothetical protein
**KASP5.128**	MCINX15280	10	scaffold7964	13465	Candidate	

**KASP5.129**	MCINX15572	17	scaffold825	253147	Candidate	Hexamerin 2, Hexamerin 4
**KASP5.130**	MCINX16047	20	scaffold889	27903	Candidate	
**KASP5.133**	MCINX16177	15	scaffold903	70120	Candidate	
**KASP5.135**	MCINX16461	3	scaffold949	27424	Candidate	Glucose-regulated protein 78, Glucose-regulated protein 78, putative
**KASP5.23**	MCINX1575	11	scaffold1376	75299	Candidate	Cad74A
**KASP5.30**	MCINX3416	21	scaffold1729	108053	Candidate	Renilla-luciferin 2-monooxygenase
**KASP5.4**	MCINX211	2	scaffold1064	144949	Candidate	Conserved hypothetical protein, Cuticular protein 3, RR-2 family
**KASP5.40**	MCINX5469	9	scaffold2061	150910	Candidate	
**KASP5.66**	MCINX6084	3	scaffold2210	15010	Candidate	Glypican
**KASP5.7**	MCINX217	2	scaffold1064	192234	Candidate	CPR64, Cuticular protein RR-2 motif 63
**KASP5.85**	MCINX8356	2	scaffold2881	7795	Candidate	Nop58 protein, Nop58
**KASP5.99**	MCINX9814	24	scaffold3502	186790	Candidate	Myostatin variant A (Fragment), Myostatin (Fragment)
**KASP6.1**	MCINX132	5	scaffold1048	134192	Candidate	GstD1
**KASP6.10**	MCINX401	16	scaffold1104	35389	Candidate	Chemosensory protein 2 variant (Fragment), Chemosensory protein 2 (Fragment)
**KASP6.102**	MCINX6004	26	scaffold2185	44668	Candidate	Hypothetical protein, Hypothetical protein
**KASP6.12**	MCINX472	7	scaffold1129	26699	Candidate	Calcineurin B (Fragment), Calcineurin B homologous protein 2
**KASP6.137**	MCINX8377	6	scaffold288	77436	Candidate	MDL2 (Fragment)
**KASP6.140**	MCINX8815	1	scaffold304	89696	Candidate	Paramyosin (Fragment), GA19246-PA
**KASP6.147**	MCINX10130	25	scaffold3657	58041	Candidate	Hypothetical protein
**KASP6.156**	MCINX10594	21	scaffold391	6515	Candidate	Hemocyanin (Fragment), AAEL006874
**KASP6.164**	MCINX10890	5	scaffold413	11132	Candidate	Chorion b-ZIP transcription factor

**KASP6.194**	MCINX13403	21	scaffold5866	4387	Candidate	Prophenoloxidase subunit 1, Pro-phenol oxidase
**KASP6.218**	MCINX14039	16	scaffold658	68226	Candidate	Cellular nucleic acid-binding protein, CNBP
**KASP6.247**	MCINX15884	6	scaffold872	25905	Candidate	Protein tyrosine phosphatase type IVA 2, Protein tyrosine phosphatase type IVA, me
**KASP6.260**	MCINX6277	2	scaffold2264	41130	Candidate	Noc2l protein, Noc2l protein
**KASP6.261**	MCINX11173	9	scaffold427	239882	Candidate	Sterile alpha and TIR motif-containing protein 1 (Fragment), Sterile alpha and TIR mo
**KASP6.263**	MCINX11277	15	scaffold433	19530	Candidate	Vesicle-associated membrane protein 7
**KASP6.265**	MCINX12915	19	scaffold5307	4125	Candidate	Potassium intermediate/small conductance calcium-activated channel, subfamily N,
**KASP6.49**	MCINX2443	16	scaffold1548	289001	Candidate	Quinoid dihydropteridine reductase
**KASP7.112**	NA	2	scaffold2086	49305	Neutral	Not annotated
**KASP7.113**	NA	15	scaffold2089	90213	Neutral	Not annotated
**KASP7.127**	NA	18	scaffold2424	16092	Neutral	Not annotated
**KASP7.16**	NA	12	scaffold1067	20731	Neutral	Not annotated
**KASP7.164**	NA	21	scaffold2615	52983	Neutral	Not annotated
**KASP7.166**	NA	5	scaffold2746	2454	Neutral	Not annotated
**KASP7.202**	NA	6	scaffold2850	40949	Neutral	Not annotated
**KASP7.258**	NA	24	scaffold3039	6076	Neutral	Not annotated
**KASP7.277**	NA	26	scaffold3200	6709	Neutral	Not annotated
**KASP7.283**	NA	28	scaffold3280	10986	Neutral	Not annotated
**KASP7.310**	NA	13	scaffold3563	36552	Neutral	Not annotated
**KASP7.337**	NA	10	scaffold382	81301	Neutral	Not annotated
**KASP7.360**	NA	16	scaffold4270	52086	Neutral	Not annotated

**KASP7.361**	NA	16	scaffold4286	9342	Neutral	Not annotated
**KASP7.374**	NA	22	scaffold444	104441	Neutral	Not annotated
**KASP7.391**	NA	29	scaffold4661	46991	Neutral	Not annotated
**KASP7.454**	NA	19	scaffold5338	8223	Neutral	Not annotated
**KASP7.456**	NA	23	scaffold5357	12554	Neutral	Not annotated
**KASP7.467**	NA	17	scaffold546	53771	Neutral	Not annotated
**KASP7.486**	NA	1	scaffold70	16568	Neutral	Not annotated
**KASP7.488**	NA	7	scaffold7393	34989	Neutral	Not annotated
**KASP7.499**	NA	14	scaffold7875	64448	Neutral	Not annotated
**KASP7.512**	NA	3	scaffold835	43108	Neutral	Not annotated
**KASP7.527**	NA	3	scaffold9635	4996	Neutral	Not annotated
**KASP7.53**	NA	4	scaffold1337	10523	Neutral	Not annotated
**KASP7.76**	NA	1	scaffold1777	7191	Neutral	Not annotated
**KASP7.81**	NA	25	scaffold1799	11328	Neutral	Not annotated
**KASP7.83**	NA	11	scaffold1810	50056	Neutral	Not annotated
**KASP7.84**	NA	22	scaffold1826	3522	Neutral	Not annotated
**KASP7.97**	NA	6	scaffold2003	17223	Neutral	Not annotated
**KASP8.131**	MCINX4001	23	scaffold182	258698	Gap filling	
**KASP8.148**	MCINX4424	15	scaffold1892	48979	Gap filling	Hypothetical protein, Hypothetical protein
**KASP8.161**	MCINX4529	11	scaffold1908	49848	Gap filling	Charged multivesicular body protein 1b (Fragment)
**KASP8.166**	MCINX4549	29	scaffold1912	19645	Gap filling	Limulus clotting factor C, Complement C1r/s-A isotype

**KASP8.174**	MCINX4849	29	scaffold1961	6233	Gap filling	Arid5b
**KASP8.183**	MCINX5204	2	scaffold2021	129213	Gap filling	Alsin
**KASP8.202**	MCINX5649	18	scaffold2103	16193	Gap filling	V-cath
**KASP8.223**	MCINX5923	12	scaffold2169	13044	Gap filling	Cdk2
**KASP8.245**	MCINX6466	29	scaffold2316	4099	Gap filling	
**KASP8.251**	MCINX6468	30	scaffold2317	42722	Gap filling	
**KASP8.252**	MCINX6486	12	scaffold2322	131346	Gap filling	Probable DNA topoisomerase 2, Top2
**KASP8.262**	MCINX7131	11	scaffold2532	1325	Gap filling	RNA splicing factor (Pad-1), putative, CG11266<up>f05171</up&gt
**KASP8.274**	MCINX7518	13	scaffold2649	52955	Gap filling	4-hydroxyphenylpyruvate dioxygenase (Fragment), Translocation protein 1 (Fragmen
**KASP8.285**	MCINX7865	19	scaffold2710	13855	Gap filling	GstD1, GstD1
**KASP8.287**	MCINX7983	22	scaffold2736	5203	Gap filling	BRAFLDRAFT_200682
**KASP8.313**	MCINX9024	28	scaffold3152	77036	Gap filling	Hypothetical protein, CG3541
**KASP8.315**	MCINX9038	22	scaffold3157	76848	Gap filling	Glycosyl hydrolase, family 31/fibronectin type III domain protein, Zgc:123172
**KASP8.339**	MCINX9754	25	scaffold3483	14528	Gap filling	Sugar transporter 4, GE20094
**KASP8.342**	MCINX9895	5	scaffold3553	16575	Gap filling	TGF-beta receptor type-1, Activin A receptor, type IIA precursor
**KASP8.345**	MCINX9936	25	scaffold3564	107947	Gap filling	Tubulin, beta 2C, Beta-Tub56D
**KASP8.35**	MCINX717	30	scaffold1184	75784	Gap filling	UPF0767 protein C1orf212, UPF0767 protein C1orf212 homolog
**KASP8.397**	MCINX11988	25	scaffold4723	29643	Gap filling	ATP-dependent RNA helicase
**KASP8.403**	MCINX12294	7	scaffold4875	43690	Gap filling	
**KASP8.406**	MCINX12436	31	scaffold4943	5626	Gap filling	
**KASP8.414**	MCINX12641	19	scaffold510	130737	Gap filling	MAP1B

**KASP8.42**	MCINX836	6	scaffold1209	195467	Gap filling	33.6 kDa small heat shock protein
**KASP8.422**	MCINX12774	12	scaffold5194	33610	Gap filling	CG11247, CG11247
**KASP8.44**	MCINX1037	10	scaffold1257	42135	Gap filling	Hypothetical protein, Signal recognition particle 19 kDa protein (Fragment)
**KASP8.447**	MCINX13015	4	scaffold541	47422	Gap filling	60S ribosomal protein L44, 60S ribosomal protein L44
**KASP8.48**	MCINX1088	27	scaffold1271	7571	Gap filling	Putative zinc finger protein 709
**KASP8.499**	MCINX15088	20	scaffold776	66911	Gap filling	CG4042, CG4042
**KASP8.519**	MCINX15641	24	scaffold83	12865	Gap filling	Sugar transporter ERD6-like 5, Sugar transporter ERD6-like 5
**KASP8.522**	MCINX15638	14	scaffold839	133539	Gap filling	Guanine nucleotide-binding protein subunit beta-like protein 1, Guanine nucleotide-bi
**KASP8.537**	MCINX15847	20	scaffold867	13984	Gap filling	Glucosyl/glucuronosyl transferases
**KASP8.82**	MCINX2560	10	scaffold1567	47209	Gap filling	Hypothetical protein, Hypothetical protein
**KASP8.92**	MCINX2713	14	scaffold1597	75402	Gap filling	CG7630, CG7630
**KASP8.96**	MCINX3055	24	scaffold1660	123146	Gap filling	Probable prefoldin subunit 4, Probable prefoldin subunit 4
**KASP8.53**	MCINX1516	21	scaffold1361	29905	Gap filling	Hypothetical protein, CCR4-NOT transcription complex, subunit 10
**KASP2.118**		NA	scaffold1524	25351	Candidate	Not annotated
**KASP2.121**	MCINX1487	15	scaffold1353	15434	Candidate	Coatamer protein, beta subunit, putative, TpnT
**KASP2.75**	MCINX13667	NA	scaffold6141	8874	Candidate	RAD6 homolog B, RAD6 homolog B
**KASP3.103**	MCINX8066	12	scaffold2758	49413	Candidate	L(1)G0431
**KASP3.104**	MCINX8106	11	scaffold2794	12176	Candidate	Transcription elongation factor 1 homolog, Transcription elongation factor 1 homolog
**KASP3.13**	MCINX595	9	scaffold1158	4351	Candidate	Hypothetical protein, Receptor-type tyrosine-protein phosphatase N2
**KASP3.141**	MCINX9374	25	scaffold3283	18361	Candidate	Glucose-6-phosphate isomerase (Fragment)
**KASP3.142**	MCINX9374	25	scaffold3283	24775	Candidate	Glucose-6-phosphate isomerase (Fragment)

**KASP3.153**	MCINX11169	9	scaffold427	226034	Candidate	Hypothetical protein
**KASP3.164**	MCINX12636	4	scaffold5108	14248	Candidate	CG13392, Dmel_CG13392
**KASP3.165**	MCINX12882	11	scaffold528	18649	Candidate	Cuticular protein RR-2 motif 83, Cuticular protein CPR99
**KASP3.167**	MCINX13215	3	scaffold563	245306	Candidate	Nicastrin (Fragment), CG7012
**KASP3.178**	MCINX14336	12	scaffold693	51700	Candidate	Probable 60S ribosomal protein L12, Probable 60S ribosomal protein L12
**KASP3.187**	MCINX15717	27	scaffold857	68155	Candidate	Pro-resilin (Fragment)
**KASP3.190**	MCINX16170	6	scaffold902	84229	Candidate	
**KASP3.191**	MCINX16177	15	scaffold903	71163	Candidate	
**KASP3.3**	MCINX49	31	scaffold1020	65472	Candidate	
**KASP3.32**	MCINX2499	19	scaffold1559	151801	Candidate	Succinyl-CoA ligase [ADP-forming] beta-chain, mitochondrial
**KASP3.44**	MCINX3958	6	scaffold1825	57040	Candidate	Cuticular protein RR-1 motif 1, Cuticular protein RR-1 motif 1
**KASP3.7**	MCINX246	21	scaffold1070	141012	Candidate	Probable dolichyl pyrophosphate Man9GlcNAc2 alpha-1, 3-glucosyltransferase
**KASP3.70**	MCINX6050	1	scaffold21	34848	Candidate	CG1600, CG1600
**KASP5.10**	MCINX947	18	scaffold1237	30279	Candidate	Hypothetical protein, Hypothetical protein
**KASP5.20**	MCINX1574	11	scaffold1376	66579	Candidate	Alpha-esterase, Putative odorant-degrading enzyme
**KASP5.29**	MCINX2989	2	scaffold1641	22707	Candidate	Recombining binding protein suppressor of hairless, Recombining binding protein su
**KASP5.37**	MCINX4639	23	scaffold1926	58619	Candidate	Hypothetical protein
**KASP5.8**	MCINX452	11	scaffold1124	64208	Candidate	TRM5 tRNA methyltransferase 5 homolog, TRNA (guanine) methyltransferase Trm5
**KASP5.86**	MCINX8663	16	scaffold29	34816	Candidate	Threonyl-tRNA synthetase, cytoplasmic, putative, SJCHGC03807 protein
**KASP5.88**	MCINX8641	14	scaffold2985	10876	Candidate	Hypothetical protein
**KASP5.91**	MCINX9107	9	scaffold3185	3464	Candidate	Hypothetical protein, Hypothetical protein

**KASP6.113**	MCINX6479	27	scaffold231	19259	Candidate	
**KASP6.225**	MCINX14648	14	scaffold730	7670	Candidate	Moricin B3, Moricin B3
**KASP6.39**	MCINX1751	3	scaffold1413	21670	Candidate	Thioredoxin-dependent peroxide reductase 1, Tsa1p
**KASP6.57**	MCINX3156	2	scaffold1671	96854	Candidate	Mitochondrial fission 1 protein, Mitochondrial fission 1 protein
**KASP7.145**		26	scaffold2497	108837	Neutral	Not annotated
**KASP7.315**		13	scaffold3659	48449	Neutral	Not annotated
**KASP7.321**		28	scaffold3685	41884	Neutral	Not annotated
**KASP7.324**		30	scaffold3693	14961	Neutral	Not annotated
**KASP7.417**		24	scaffold4893	53260	Neutral	Not annotated
**KASP7.469**		12	scaffold552	117110	Neutral	Not annotated
**KASP8.138**	MCINX4022	21	scaffold1834	66590	Gap filling	NADH dehydrogenase [ubiquinone] 1 alpha subcomplex subunit 7 (Fragment), Putat
**KASP8.139**	MCINX4200	28	scaffold1865	271957	Gap filling	
**KASP8.240**	MCINX6197	19	scaffold2240	7925	Gap filling	Dolichol-phosphate mannosyltransferase subunit, putative
**KASP8.301**	MCINX8630	5	scaffold297	34030	Gap filling	Glutathione S-transferase sigma, GST-like hemolymph protein
**KASP8.37**	MCINX771	17	scaffold119	228038	Gap filling	Fatty-acid amide hydrolase 2, Putative amidotransferase subunit A
**KASP8.72**	MCINX2582	5	scaffold156	61480	Gap filling	Putative myosin regulatory light chain 2 smooth muscle, Hypothetical protein

SI References

1. T. Schulz, J. Vanhatalo, M. Saastamoinen, Long-term demographic surveys reveal a consistent relationship between average occupancy and abundance within local populations of a butterfly metapopulation. *Ecography* **43**, 306–317 (2020).

2. G.-A. Fuglstad, D. Simpson, F. Lindgren, H. Rue, Constructing Priors that Penalize the Complexity of Gaussian Random Fields. *J. Am. Stat. Assoc.* **114**, 445–452 (2019).

3. A. P. Dawid, Present Position and Potential Developments: Some Personal Views: Statistical Theory: The Prequential Approach. *J. R. Stat. Soc. Ser. Gen.* **147**, 278 (1984).

4. C. Czado, T. Gneiting, L. Held, Predictive Model Assessment for Count Data. *Biometrics* **65**, 1254–1261 (2009).

5. O.-P. Smolander, *et al.*, Improved chromosome-level genome assembly of the Glanville fritillary butterfly (*Melitaea cinxia*) integrating Pacific Biosciences long reads and a high-density linkage map. *GigaScience* **11**, giab097 (2022).

6. I. R. Franklin, The distribution of the proportion of the genome which is homozygous by descent in inbred individuals. *Theor. Popul. Biol.* **11**, 60–80 (1977).

7. J. F. Crow, M. Kimura, *An introduction to population genetics theory*, Indian ed (Scientific Publisher (India) ; The Blackburn Press, 2010).
